# Hindlimb kinematics, kinetics and muscle dynamics during sit-to-stand and sit-to-walk transitions in emus (*Dromaius novaehollandiae*)

**DOI:** 10.1242/jeb.247519

**Published:** 2024-12-02

**Authors:** Yuting Lin, Jeffery W. Rankin, Luís P. Lamas, Mehran Moazen, John R. Hutchinson

**Affiliations:** ^1^ Structure and Motion Laboratory, Department of Comparative Biomedical Sciences, Royal Veterinary College, Hatfield AL9 7TA, UK; ^2^ Pathokinesiology Laboratory, Rancho Los Amigos National Rehabilitation Center, Downey, CA 90242, USA; ^3^ CIISA, Faculty of Veterinary Medicine, University of Lisbon, Lisbon 1300-477, Portugal; ^4^ Department of Mechanical Engineering, University College London, London WC1E 7JE, UK

**Keywords:** Emu, Sit-to-stand, Sit-to-walk, Musculoskeletal simulation, Inverse dynamics, OpenSim

## Abstract

Terrestrial animals not only need to walk and run but also lie prone to rest and then stand up. Sit-to-stand (STS) and sit-to-walk (STW) transitions are vital behaviours little studied in species other than humans so far, but likely impose biomechanical constraints on limb design because they involve near-maximal excursions of limb joints that should require large length changes and force production from muscles. By integrating data from experiments into musculoskeletal simulations, we analysed joint motions, ground reaction forces, and muscle dynamics during STS and STW in a large terrestrial, bipedal and cursorial bird: the emu (*Dromaius novaehollandiae*; body mass ∼30 kg). Simulation results suggest that in both STS and STW, emus operate near the functional limits (∼50% of shortening/lengthening) of some of their hindlimb muscles, particularly in distal muscles with limited capacity for length change and leverage. Both movements involved high muscle activations (>50%) and force generation of the major joint extensor muscles early in the transition. STW required larger net joint moments and non-sagittal motions than STS, entailing greater demands for muscle capacity. Whilst our study involves multiple assumptions, our findings lay the groundwork for future studies to understand, for example, how tendon contributions may reduce excessive muscle demands, especially in the distal hindlimb. As the first investigation into how an avian species stands up, this study provides a foundational framework for future comparative studies investigating organismal morphofunctional specialisations and evolution, offering potential robotics and animal welfare applications.

## Teaser

**Summary:** The dynamics, biomechanical constraints and musculotendinous coordination strategies during the sit-to-stand/walk transitions for a large bipedal bird – the emu – inform morphology, evolution and potential robotic applications.

## INTRODUCTION

The abilities to perform sit-to-stand (STS) and sit-to-walk (STW) behaviours are fundamental for humans (e.g. Aissaoui and Dansereau, 1999; Sloot et al., 2020; Smith et al., 2020) and terrestrial animals (e.g. Brouwers et al., 2023; Ellis et al., 2018; Gardner, 2011; Lidfors, 1989). These behaviours must overcome gravitational constraints to substantially elevate the body's centre of mass (COM) from a flexed initial limb posture, likely resulting in large joint moments and potentially unfavourable effective mechanical advantage (EMA) (Biewener, 1989). In humans, both STS and STW require considerable muscle strength and coordination for task execution and balance control (Dehail et al., 2007; Doorenbosch et al., 1994; Ellis et al., 1984; Riley et al., 1997; Roebroeck et al., 1994; Schultz et al., 1992; Yoshioka et al., 2009). In older adults, these activities even approach the upper limits of muscle capacity (Hortobágyi et al., 2003; Hughes, 1996). However, despite extensive studies on movement patterns and muscle recruitment (Hughes et al., 1994; Perera et al., 2023; Smith et al., 2020), understanding of the control strategies used during these movements remains elusive even for humans (e.g. Actis et al., 2018; Bobbert et al., 2016; Pandy et al., 1995; Shia et al., 2018). Remarkably, research on the biomechanics of STS and STW transitions (henceforth, simply STS and STW) in animals is extremely scarce, with only three studies on dogs as examples (Ellis et al., 2018; Feeney et al., 2007; Triviño et al., 2024).

Birds, especially large, cursorial species, including emus, present a unique opportunity to understand limb structure and locomotor function in both extant and extinct species (e.g. Carrano, 1999). Large, cursorial bird species are known for their remarkable speeds and efficient locomotion as a result of elevated storage and release of elastic energy in tendons, with muscle fibres predicted to act either approximately isometrically or slowly shortening (Badri-Spröwitz et al., 2022; Rankin et al., 2016; Rubenson et al., 2011; Smith and Wilson, 2013). These simulated fibre actions during locomotion are also consistent with studies of *in vivo* muscle function in other species (e.g. Biewener, 1998; Daley and Biewener, 2003; Fukunaga et al., 2001; Lichtwark et al., 2007; Lichtwark and Wilson, 2006; Roberts et al., 1998). However, when compared with other forms of locomotion, STS and STW impose unique musculoskeletal demands because they require potentially large joint moments at postures with a low strength-to-weight ratio. In particular, the challenges faced by cursorial birds during STS and STW, including substantial fibre length change and force production, are probably compounded by their elongated, flexed limbs and specialised muscular configurations (e.g. allometrically shorter muscle fibres in distal limbs) (Biewener, 2005; Bishop et al., 2021d; Dick and Clemente, 2017; Lamas et al., 2014; Maloiy et al., 1979). Understanding the muscle–tendon dynamics during STS and STW in cursorial birds should provide valuable insights into the biomechanical constraints and allow for investigation into how non-locomotor movements shape locomotor form and function.

**Fig. 1.**
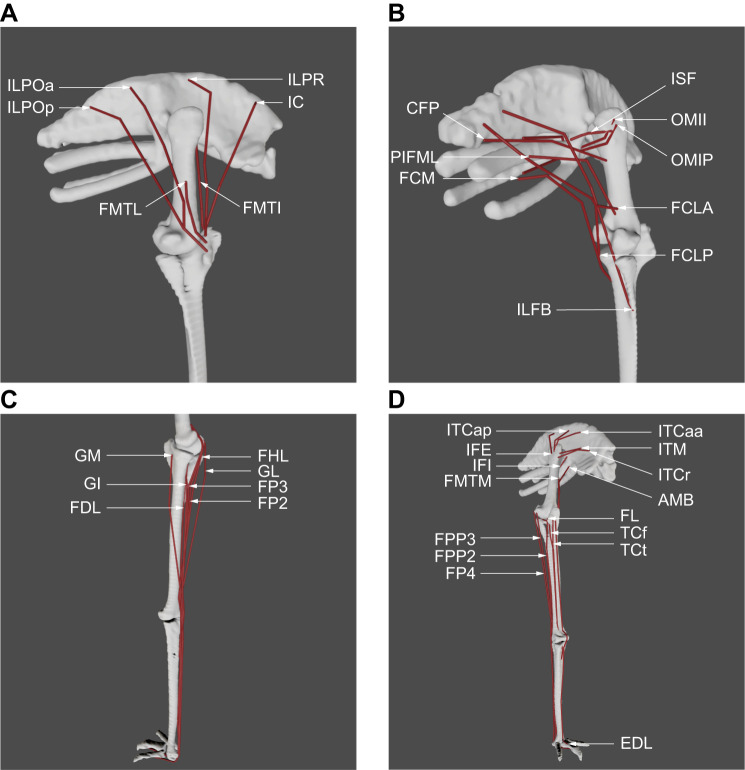
**Muscle groups included in the musculoskeletal model of the right hindlimb of the representative emu, in the neutral pose with initial muscle attachments used**. See Table 1 for full muscle names. (A) ‘Triceps femoris’ knee extensor muscles (except AMB, FMTM), in lateral view. (B) OMII, OMIP, ‘hamstring’ (FCM, FCLP, FCLA) thigh muscles, and other caudally positioned pelvic muscles in caudolateral view. (C) Distal hindlimb muscles on the plantar (caudal) surface of the limb, in medial view. (D) Deep dorsal (IFE, ITCaa, ITCap, ITCr, ITM, IFI), AMB and distal hindlimb muscles on the dorsal (cranial) surface of the limb, in craniolateral view.

**Fig. 2.**
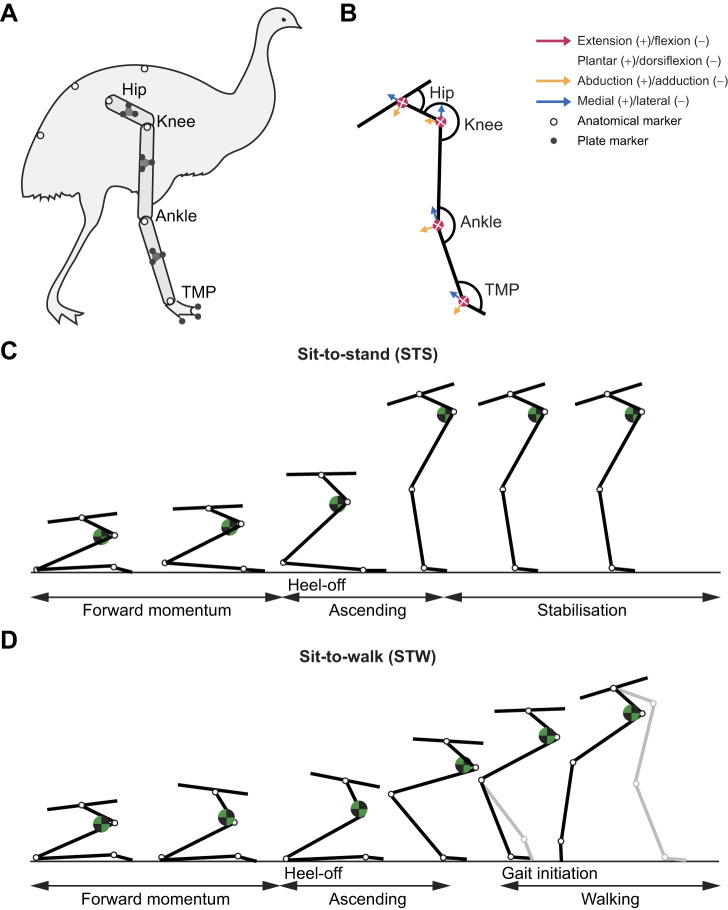
**Schematic of emu hindlimb marker locations, joint axes definitions, and sit-to-stand (STS) and sit-to-walk (STW) cycles.** (A) Emu hindlimb marker locations. (B) Joint axes definitions [extension/flexion, plantar/dorsiflexion, abduction/adduction, and lateral (external) or medial (internal) rotation]. (C) STS and (D) STW cycles. The foot position affects how horizontally close the COM (green and black circle) is to the plantar/caudal edge of the third tarsometatarsophalangeal (TMP) joint. Heel-off in STS and STW are denoted. Gait initiation in STW occurs at the end of the ascending phase and is denoted by the toe-off of the swing-phase foot.

**Fig. 3.**
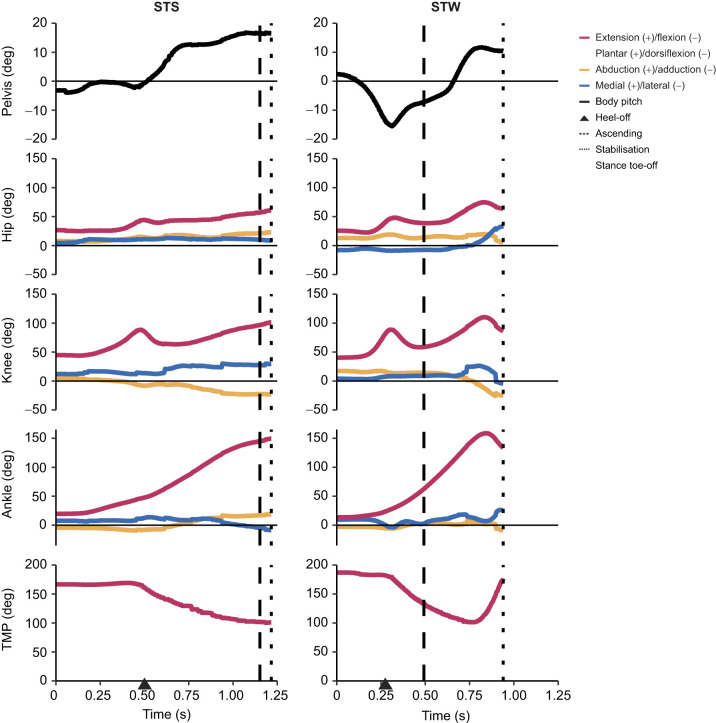
**Body and hindlimb joint angles during STS and STW in the exemplar trials.** See Fig. 1 for marker/angle definitions. For STW, the results were derived from the stance leg. (A) STS and (B) STW events and phases are denoted, where heel-off is represented by an arrow, the end of the ascending phase is represented by a dashed line, and the end of the stabilisation phase of STS and walking phase of STW are represented by dotted lines.

This study investigates the movement dynamics, biomechanical constraints and musculotendinous coordination strategies during STS and STW in emus (*Dromaius novaehollandiae*). Emus serve as an ideal avian model because of their cursorial adaptations, manageability and limb structure, and they offer a compelling basis for comparisons with humans, as shown by the similarities in walking biomechanics (Goetz et al., 2008). Our objectives are twofold: firstly, to quantify the patterns of hindlimb kinematics and kinetics in emu STS and STW behaviours, and secondly, to identify the mechanical constraints and possible musculotendinous coordination strategies used by emus in performing the two tasks. We hypothesised that: (1) both transitions would require high muscle activations in the key hip, knee, and ankle extensor muscles and in other muscles whose primary actions are non-parasagittal (Lamas et al., 2014; and Fig. 1); (2) emu hindlimb muscles would operate near their functional limits, especially for distal muscles (i.e. muscles crossing the ankle and TMP joints) (Lamas et al., 2014; Fig. 1); (3) hindlimb tendons would play important roles in preventing large muscle activations and length changes during the movements; and (4) STW in emus would entail greater demands for muscle capacity than STS.

**Table 1.**
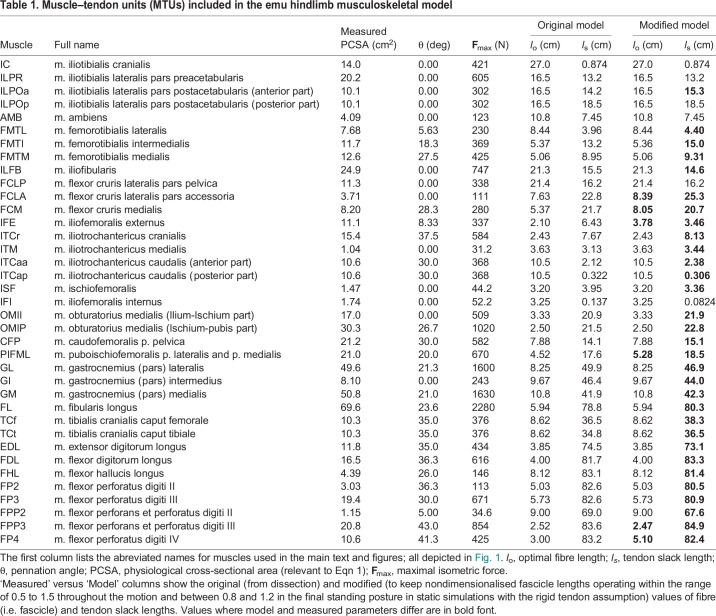
Muscle–tendon units (MTUs) included in the emu hindlimb musculoskeletal model

**List of symbols and abbreviations**

*a*
: simulated muscle activation
BOS: base of support
BW: body weight
COM: centre of mass
COP: centre of pressure
CT: computed tomography
DOF: degrees of freedom
EMA: effective mechanical advantage
**F**_max_
: muscle maximal isometric force
**F**_m_ *
: body weight-nondimensionalised muscle force
GRF: ground reaction force
L: anatomical leg length
l*
: nondimensionali​se​d fibre length
*l*_o_
: optimal fibre length
*l*_s_
: tendon slack length
*m*
: muscle belly mass
ML: medio-lateral
MTU: muscle–tendon unit
ROM: range of motion
STS: sit-to-stand
STW: sit-to-walk
TMP: tarsometatarsophalangeal
σ: muscle maximum isometric stress
ρ: muscle tissue density

See Table 1 for a list of abbreviations and full names of all muscles analysed.

## MATERIALS AND METHODS

### Overview

The study comprises four primary stages, described in detail below. First, we collected STS and STW kinematic and kinetic data from two subadult male emus [*Dromaius novaehollandiae* (Latham 1790), each with a body mass ∼30 kg]. We then combined these empirical data into a detailed musculoskeletal model of an emu hindlimb, scaled to the two subjects, to estimate hindlimb net joint moments using OpenSim's inverse dynamics routine (Delp et al., 2007). To compare emu STS and STW, we defined phases marked by distinct events (below) and focused primarily on the onset of the movements until standing upright or initiating gait. Following this, we used the calculated joint moments to estimate the required muscle activations, nondimensionali​se​d fibre lengths (l*) and muscle forces by conducting dynamic optimisation simulations (henceforth, simply dynamic simulations) with the incorporation of full tissue properties in OpenSim Moco (Dembia et al., 2020). In addition, we performed static optimisation simulations (henceforth, simply static simulations) with OpenSim (Delp et al., 2007), which assumed rigid tendons, and conducted sensitivity analyses of tendon slack lengths (*l*_s_) (detailed below). By comparing the simulated muscle activations, forces and length changes from two simulation frameworks, we aimed to test whether muscle fibres alone could meet the joint moment requirements and how limited they would be in doing so (our first two hypotheses) as well as our hypothesis on the roles of tendons. To control for potential confounding factors – such as time coupling and different muscle and tendon models used in different programmes – we also generated additional simulations in OpenSim Moco assuming rigid tendons, similarly to our static optimisation simulations. Detailed procedures regarding the collection of experimental data, development of the musculoskeletal model and application of optimisation frameworks are detailed below.

### Animals

Four emus were involved in the study, including one emu cadaver (male; body mass 48.8 kg) used for musculoskeletal model development (Lamas, 2015), and three emus trained to complete the STS and STW tasks; however, only two of the three birds were compliant with the procedures necessary for data collection. Data from the two emus were used to characterise STS and STW kinematics and kinetics, and two exemplar trials (one for each task) from one emu were used in musculoskeletal simulations (below). All emus were hatched at a commercial breeding farm in the UK and subsequently reared from 4 weeks of age at the Royal Veterinary College. Their diet consisted primarily of a commercial ostrich pelleted diet supplemented with grass. From 6 weeks of age, they had unrestricted access to both commercial food and grass. At 24 weeks, their diet transitioned from an ostrich grower diet to adult ostrich pelleted food (Dodson and Horrel Ltd, Kettering, Northamptonshire, UK). Throughout their development, no constraints were placed on their regular exercise regimen, and all emus had equal access to exercise areas and conditions. All animals were euthanised after the completion of other experimental procedures using a lethal intravenous injection of a barbiturate, following the induction of deep terminal general anaesthesia with intramuscular injection of ketamine and xylazine. The carcass of the emu used for model development was stored in a −20°C freezer before dissection. Initial data were obtained shortly after euthanasia, while a small subset of data, such as the ISF, became available only 2 years later. Dissection began within 4 days of removal from the freezer to allow for thawing. All dissections were completed within a 6 week period and led by the same individual (L.P.L.). Approval for all studies involving these animals was obtained from the Royal Veterinary College's Ethics and Welfare Committee, following the Animals (Scientific Procedures) Act 1986 under a Home Office license number 70/7122.

### Experimental data

Data collection occurred indoors at the Royal Veterinary College's Structure and Motion Lab. The two subadult male emus used in data collection weighed ∼27.4 and ∼28.5 kg. These birds were generally settled and used to the experimental environment. If not stimulated, birds would normally sit without incentive. Once sitting, gentle stimuli (noise or touching them) were used for them to naturally stand up again. Prioritising the avoidance of fatigue effects, a resting period of 1–5 min between consecutive trials was allowed for each bird. Throughout the study, the birds had access to ample rest, companionship, water and food. In total, we performed approximately 30 trials for each of the three individuals, resulting in 39 successfully recorded STS and STW trials. In all successfully recorded trials, the birds started in a near-symmetrical, crouched position. We classified trials where minimal forward movement occurred upon standing upright as STS according to Riley et al. (1991) and defined trials that involved at least one complete stride forward during rising as STW per Perera et al. (2023).

Emus standing up from a sitting position exhibited patterns akin to humans rising from a chair, where the ankle touching the ground in emus corresponds to the buttocks on a chair during the sitting position in humans. Therefore, we adopted a similar phase delineation as used in human chair-rising studies (Kralj et al., 1990; Schenkman et al., 1990) for defining STS phases in emus (Fig. 2C).

(1) Forward momentum phase: this phase involved the generation of linear forward momentum through an initial forward rotation of the trunk and pelvis into flexion (or downward pitch), identified by movement onset [marked by a 5% change in the vertical ground reaction force (GRF)] and concluded when the tarsometatarsus lifted off from the ground (marked by a 5 mm change in the ankle marker height). Owing to the more horizontal body posture and rigid trunk in emus, trunk flexion is less pronounced compared with humans. Therefore, the term ‘forward momentum’ is used instead of ‘flexion momentum’ for clarity.

(2) Ascending phase: initiated at heel-off and ended upon standing upright or initiating gait; as assessed by a root mean squared (RMS) cost function, defined as the square root of the sum of the squares of the differences between observed and expected GRFs (body weight for vertical GRF and zero for anterior-posterior GRF), with RMS values minimised to be within a specified plausible range. An RMS tolerance level of 0.01 was used to ensure robustness to minor fluctuations around the minimum cost value.

(3) Stabilisation phase: started at the end of the ascending phase and ended when the vertical GRF equalled body weight and fluctuations of 0.1% – characteristic of quiet standing – were detected.

STW phases were similar to STS phases. Since STW in emus was a fluid transition from STS to gait, we defined the first two phases of STW using the identical events as those in STS. The walking phase started at gait initiation – denoted as the toe-off of the swing-phase (ipsilateral) foot – and ended at the toe-off of the stance-phase (contralateral) foot (Fig. 2D).

Coordinate data collected from retro-reflective marker clusters placed on the pelvis, right-side femur, tibiotarsus, tarsometatarsus, and digits were used to compute three-dimensional (3D) segment and joint kinematics (Fig. 2A; marker set definitions in Supplementary Materials and Methods). Anatomical landmarks were identified through palpation and visual inspection, and markers were attached to the skin using hair extension glue and double-sided tape after careful feather clipping in these areas. Additional feathers, including some wing feathers, were also trimmed to ensure marker visibility throughout the experiment. Feather trimming was restricted to areas around the hip and knee, where the wings naturally cover, minimising potential alterations that could provoke pecking. Continuous monitoring for signs of stress or aggression was conducted to ensure the well-being of the emus. Marker data were recorded at 250 Hz using 10 Oqus cameras (Qualisys Motion Capture Systems v 2.6.673, Gothenburg, Sweden; ±1 mm precision) and subsequently filtered (6 Hz fourth-order zero-lag low-pass Butterworth filter) to remove noise. In instances where foot markers were initially obscured, we imputed missing data with the first valid coordinate value following (Ellis et al., 2018). Using OpenSim's Inverse Kinematics routine (Delp et al., 2007), we computed joint kinematics using global optimisation, ensuring that maximum marker errors for bony landmarks were less than 2 cm and root mean square (RMS) errors were less than 1 cm (Hicks et al., 2015). Angles were initially represented as Cardan angles of rotation order *x,y*′,*z*″ relative to a neutral pose set at 0 deg (limb fully straightened). These angles were thus measured as positive or negative values relative to this pose and subsequently converted to the convention depicted in Fig. 2B. In all trials, the starting position was assumed to have zero pelvis yaw.

We recorded GRFs and centre of pressures (COPs) using Kistler force plates (Model 9287BA, Kistler Holding AG, Winterthur, Switzerland). The force data were initially collected at 500 Hz and then downsampled to 250 Hz for synchronisation with marker data. Subsequently, we conducted baseline removal and applied a filtering process using a fourth-order zero-lag low-pass Butterworth filter set at 6 Hz to refine the force data. For each trial, both emu hindlimbs were positioned on a single force plate, allowing us to measure the combined hindlimb GRF, free moment and whole-body COP data throughout the movement. Our pilot study indicated that the emus applied nearly vertical GRFs with each limb while standing up, with minimal medio-lateral (ML) GRFs during the forward momentum and ascending phases in both STS and STW, i.e. before standing upright or initiating gait (Fig. S1). To obtain single hindlimb kinetic data (GRFs, free moments and COPs) for our simulations, we assumed bilateral symmetry (i.e. half of the measured GRFs and free moments were applied to each limb) during these phases. Next, we calculated the cranio-caudal portion of the COP movement and the ML component of the third digit marker movement to create a composite, single hindlimb GRF dataset following Ellis et al. (2018). This approach ensured that the right-limb COP remained within the base of support (BOS), consisting of the right tarsometatarsus and digit segments. Finally, to apply the forces to the two segments forming the foot [i.e. the ‘tarsometatarsus’ (or ‘pes’) and the ‘digits’ segments] in the musculoskeletal model, we further partitioned the GRFs and free moments between the two segments to obtain the final GRF motion file. This partitioning was necessary because the heel also touches the ground in the sitting position; partitioning allowed realistic calculation of joint moments around the TMP joint (see Supplementary Materials and Methods for details). All data were processed in MATLAB (v2023b, MathWorks, Natick, USA).

We examined only trials where emus began in a near-symmetrical, crouched posture with both tarsometatarsus and digits on the force plate following Ellis et al. (2018), resulting in 3 STS and 9 STW trials for further analysis (see Supplementary Materials and Methods for detailed inclusion and exclusion criteria). We performed musculoskeletal simulations based on the kinematics and kinetics of two exemplar trials (one each for STS and STW) from one individual. To select the exemplar trials, qualitative assessments were made based on natural movement and data within the observed kinematic and kinetic ranges (Table S1, Figs S1 and S2, Movie 1).

### Musculoskeletal model

We constructed the musculoskeletal model by integrating muscle and tendon architecture, digitised muscle paths and computed tomography (CT) scan data obtained through dissection (Lamas, 2015). The model comprised five rigid body segments representing the right-side femur, tibiotarsus, tarsometatarsus and digits, as well as the pelvis and remainder of the body (left hindlimb omitted and body mass properties halved, assuming symmetrical support). The head, neck, trunk and (diminutive) tail of the emu were collectively modelled as a single rigid unit, consistent with prior simulation studies of avian locomotion (e.g. Bishop et al., 2021a). This approach is supported by the fact that birds exhibit a highly rigid trunk due to dorsal vertebral fusion (notarium) and a robust rib-sternal complex ventrally, and the neck and head masses relative to the body are fairly small and thus were neglected here for simplicity. Therefore, the model had 10 degrees of freedom (DOF) in the right limb representing the right hip (3 DOF), knee (3 DOF), ankle (3 DOF) and tarsometatarsophalangeal (TMP) (1 DOF) joints. In the model, the pelvis moved freely relative to the ground (3 rotational and 3 transitional DOF); however, we constrained the pelvis's roll movement, assuming bilateral symmetry during the forward momentum and ascending phases. We adjusted segment mass, inertia, length, and musculotendon length values by scaling the original model in OpenSim to match the body masses and bone lengths of the two birds used during experimental data collection (see Supplementary Materials and Methods).

Our emu hindlimb musculoskeletal model included 37 muscle–tendon unit (MTU) actuators representing 33 muscles (Fig. 1; Table 1). Actuator paths were constructed based on anatomical data using fixed points (via points) and 15 MTU wrapping objects. Each MTU actuator was represented using a Hill-type model with intrinsic force–length–velocity relationships (Millard et al., 2013). Muscle fascicle lengths; assumed to equal optimal fibre lengths (*l*_o_); masses and pennation angles were measured through dissection of the same individual used for model construction. Pennation angles refer to the angle between the muscle fibres and the line of action of the muscle. Parameters for the ISF muscle were sourced from another individual of the same body mass, because the main dissected subject lacked that muscle in good condition. The resting length of the tendon, at which passive force development is zero, varies slightly because of factors such as tissue elasticity and loading history. *l*_s_ in a Hill-type MTU model is a simplified representation of the tendon resting length, which provides a consistent reference point. Estimating *l*_s_ values is challenging owing to the complex nature of internal tendons or aponeuroses. Here, we used a numerical method in combination with manual tuning to estimate *l*_s_ values. Following assignment of architectural properties and definition of MTU paths, we estimated initial *l*_s_ for each MTU using the approach of Manal and Buchanan (2004), which computes *l*_s_ from subject-specific measures of muscle architecture and length. The calculation of *l*_s_ assumed that muscle fibres could range in length from 0.5 to 1.5 times their *l*_o_ across various behaviours including walking, STS and STW. For IFI muscle, this approach resulted in a negative *l*_s_ value. This issue likely arose from using an averaged pennation angle (i.e. a bulk pennation angle), which may have produced excessively long fibres for the MTU, thus affecting its operating range. Therefore, for this muscle, *l*_s_ was recalculated using 5% of the static trial MTU length as an initial value.

We then fine-tuned the initial *l*_s_ values to ensure nondimensionali​se​d fascicle lengths operated within the range of 0.5 to 1.5 (Hicks et al., 2015) throughout the motion and between 0.8 and 1.2 in the final standing posture in static simulations with the rigid tendon assumption; following Ellis et al. (2018). Twenty-five of the 37 muscles were adjusted, with an average *l*_s_ adjustment of 6.5% (SD 7.5%) of the originally estimated MTU length (Table 1). For some muscles, muscle fibre lengths remained unrealistic throughout the motion (i.e. <0.5 or >1.5 times resting length) even after adjustments to *l*_s_. In these instances, adjustments were made to *l*_o_ concurrently shortening or lengthening *l*_s_ to maintain a constant MTU length, to ensure that muscles operated between 0.5 and 1.5 times their optimal length. This fibre length adjustment was done for the FCLA (10% increase), FCM (50% increase), IFE (80% increase), PIFML (17% increase), FPP3 (2% decrease) and FP4 (70% increase) muscles.

Each muscle's maximal isometric force (**F**_max_) was calculated according to the formula:
(1)

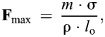
where *m* denotes muscle belly mass, σ represents maximum isometric stress of the fibres, ρ is muscle tissue density and *l*_o_ is optimal fibre length. Values of σ (300,000 N m^−2^; Medler, 2002; Hutchinson, 2004) and ρ (1060 kg m^−3^; Mendez and Keys, 1960; Hutchinson et al., 2015) that are standard for vertebrate skeletal muscle were used. Note that pennation angle was not used in Eqn 1 because OpenSim incorporates it as a separate parameter.

We included 15 additional actuators: 6 actuators to compensate for the residual forces and moments at the pelvis and 9 ‘reserve’ actuators – one for each DOF in the right limb (Hicks et al., 2015). The presence of pelvis residual forces at and moments around the pelvis was expected due to the approximations in force representations and segment characteristics of the emu. Therefore, these residual actuators were necessary to address the imbalances due to the modelling approximations. Reserve actuators were used to compensate for the potential inability of the modelled muscles to fully balance the joint torques. Our optimisation framework focused on minimising the use of these reserve actuators to ensure that required joint moments were predominantly supplied via muscles.

A list of symbols and full names for all muscles analysed is provided in Table 1.

### Simulations

For each exemplar STS/STW trial from the same individual, we used both static and dynamic simulation frameworks to estimate MTU activations, force, and length changes (detailed below). Because the appropriateness of optimisations using static versus dynamic frameworks remains a debate in the literature (Anderson and Pandy, 2001; Rankin et al., 2016; Wesseling et al., 2015; Żuk et al., 2018), we examined difference between the two approaches here to assess how these frameworks may impact conclusions about STS and STW.

The static simulation solved muscle activation patterns by optimising a pre-set objective criterion while adhering to biomechanical constraints including instantaneous force balance rather than strict static equilibrium (Delp et al., 2007). The objective criterion used here minimised the sum of squared muscle activations across all muscles at each time step. With a time step set to 0.005 s, the simulations provided time histories of MTU activations, forces and lengths throughout the movement cycle. Static simulation acts independently at each time step, excluding energy transfer between steps (e.g. tendon energy storage and return) and muscle excitation–activation dynamics, and ignores tendon compliance and passive fibre force generation. This simplified approach makes the problem-solving faster at the cost of reduced accuracy in modelling the complex, time-dependent interactions in the musculoskeletal system, potentially leading to less realistic predictions of muscle forces.

In contrast, the dynamic simulation factored in the model state from prior time steps (e.g. joint angles, muscle activation level, tendon strain) to influence the optimal solution for the current step. Linking time steps allows for the integration of muscle excitation–activation dynamics, consideration of non-rigid tendon characteristics and incorporation of passive muscle fibre force generation, making it a more realistic representation of movement mechanics. We performed dynamic simulations using the ‘MocoInverse’ tool in OpenSim Moco, which formulated the optimal control problem as a nonlinear program via direct collocation (Dembia et al., 2020), with the same objective criterion as the static optimisation routine. This setup enabled us to test the roles of tendons, as per our third hypothesis.

### Analysis

Kinematic and kinetic parameters during STS and STW were characterised using data from two emus. For kinematic values, we computed mean and standard deviation (s.d.) values using three trials of STS and three trials of STW (stance limb) from one individual (because of limited data; see Supplementary Materials and Methods). For kinetic values (i.e. GRFs and COPs), five more STW trials were included, three of which were from a second bird. For STS trials, we calculated limb joint range of motion (ROM) values from the start of the movement to the end of the stabilisation phase. For STW trials, we calculated ROM values for the stance limb, from the start of the movement to stance toe-off.

We performed a series of sensitivity analyses to test our main modelling and simulation assumptions. First, the static simulations assumed that tendons were inextensible and muscles did not generate passive forces; but *l*_s_ values could still impact our results by requiring muscle fibres to be shorter or longer. To test the robustness of this assumption, we varied *l*_s_ values by ±5% following Ellis et al. (2018), Redl et al. (2007) and Scovil and Ronsky (2006), and muscle dynamics were compared across conditions to assess how *l*_s_ changes altered them. Second, we generated additional simulations in OpenSim Moco assuming rigid tendons, similarly to static simulations. Although this approach limited forward dynamics optimisation in accounting for time-dependent muscle interactions, it reduced confounding factors such as time coupling and varying muscle and tendon models when comparing static simulations with rigid tendons to dynamic simulations in OpenSim Moco with full tissue properties. Finally, we performed dynamic simulations on two additional STS and STW trials from the same emu to examine within-individual differences.

]In evaluating the potential impact of reserve actuators on simulation outcomes (and thus how well muscles alone could generate STS and STW dynamics; our second hypothesis), we compared reserve actuator values with the net joint moments derived from OpenSim's inverse dynamics analysis following (Hicks et al., 2015). This comparison involved calculating average reserve actuator values and net joint moments and examining reserve actuator values at the instances of peak net joint moments in each DOF.

## RESULTS

### Kinematics and kinetics

#### Standing-up movements involve large ranges of joint motions

Analysis of the kinematic data revealed large ranges of joint motions in both the sagittal and non-sagittal planes in STS and STW (Fig. 3; Table S1). The ankle joint exhibited the most substantial excursion among all joints, reaching total flexion/extension ROMs of 125±5.23 deg (mean±s.d.) in STS and 134±22.9 deg in STW, extending predominantly from heel-off throughout the remainder of each motion. Emus exhibited large hip and knee joint non-sagittal motions, especially after gait initiation during STW, although non-sagittal motions were relatively small compared with sagittal motions. In addition, compared with STS, STW exhibited a shorter duration, larger peak COM velocity and smaller medio-lateral COP range during the forward momentum and ascending phases (Table S1). Overall, we found large ranges of joint motions and initially flexed limb postures in the STS and STW in emus, intimating large changes in muscle fibre lengths (below).

#### Standing-up movements require large net extensor moments around limb joints

**Fig. 4.**
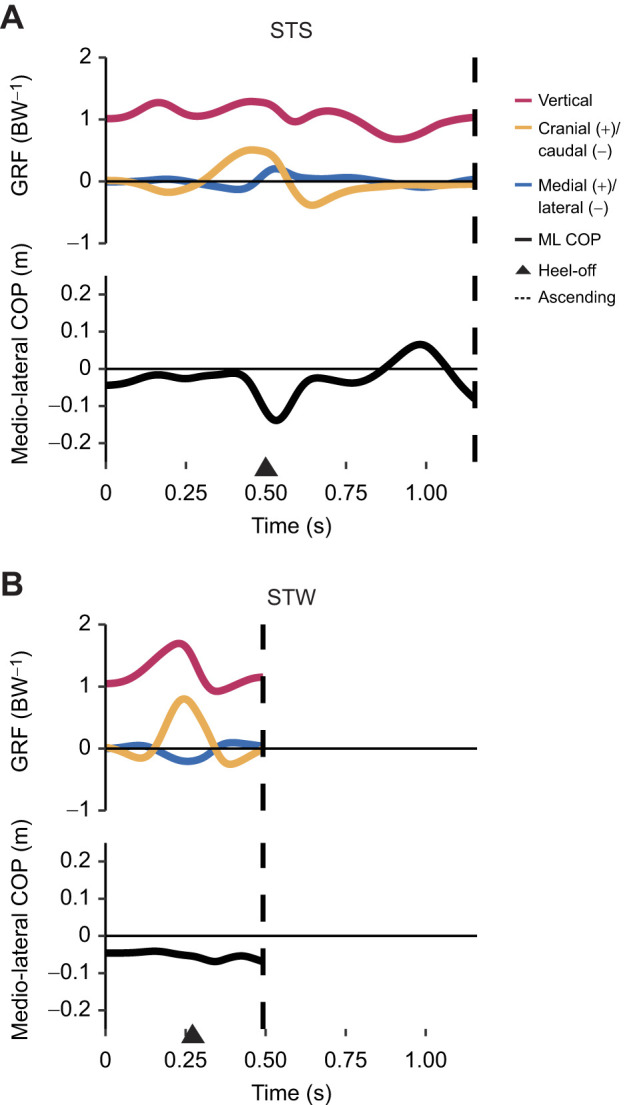
**Total (dimensionless) ground reaction forces and medio-lateral (ML) centre of pressure (COP) during STS and STW in the exemplar trials.** (A) STS and (B) STW events and phases are denoted, where heel-off is represented by an arrow and the end of the ascending phase is represented by a dashed line.

In all directions, the GRFs reached a peak early in the movements, occurring around heel-off (Fig. 4; Table S1). STW displayed a larger peak vertical GRF compared with that in STS, nearly approaching twice the body weight (BW), comparable to values reported for walking and slower speed running (Goetz et al., 2008). A second, smaller vertical GRF peak (∼1.3 BW) occurred at the onset of STS, potentially attributed to the initial adjustment of foot position closer to the COM in the exemplar trials. Despite this discrepancy, we considered the two exemplar trials (one for each behaviour) suitable for subsequent simulations owing to their natural kinematics and kinetics.

**Fig. 5.**
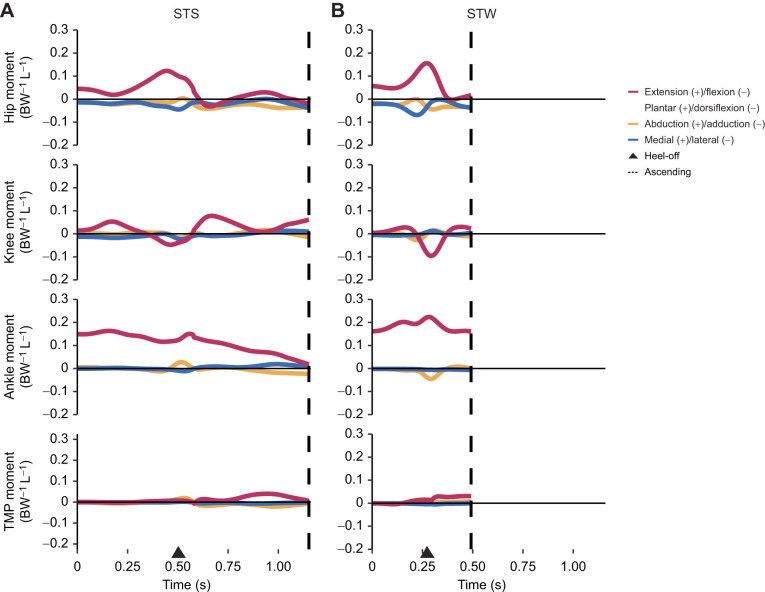
**Net (dimensionless) joint moments during STS and STW in the exemplar trials.** (A) STS and (B) STW events and phases are denoted, where heel-off is represented by an arrow and the end of the ascending phase is represented by a dashed line. For STW, the results were derived from the stance leg. The joint moments are nondimensionalised by the bird's weight (279 N, corresponding to a mass of 28.5 kg) and anatomical leg length (0.885 m). L, anatomical leg length; BW, body weight.

Consistent with the GRF results, our inverse dynamics analysis indicated a need for large net extensor moments around the hip and ankle joints and modest non-sagittal moments around the hip joint during the ascending phase, especially around heel-off (Fig. 5). The hip joint exhibited a peak extensor moment (∼0.12 dimensionless unit in STS and ∼0.15 dimensionless unit in STW) immediately before heel-off, while the ankle joint sustained a relatively large extensor moment throughout STS and STW, peaking at ∼0.16 dimensionless unit in STS transition and ∼0.22 dimensionless unit in STW right after heel-off. Although relatively small (<0.1 dimensionless unit) compared with sagittal moments, exemplar trials revealed modest non-lateral rotator and adductor moments around the hip joint during both movements, especially near heel-off. Overall, our findings indicate substantial net extensor moments during STS and STW, particularly around the ankle joint, which, due to its short-fibered distal muscles and long tendons, could potentially play a critical role in successfully executing these movements (see below).

### Muscle activation, length changes and forces

**Fig. 6.**
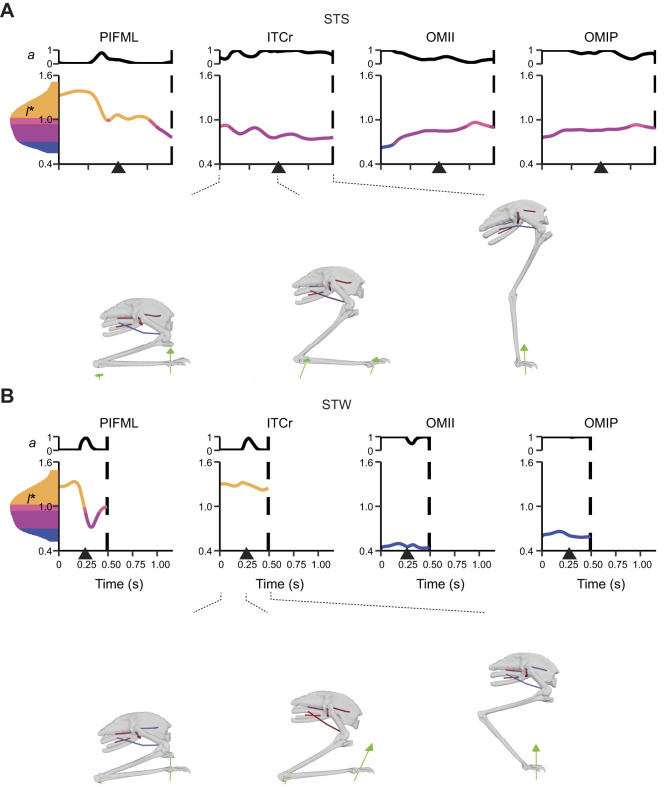
Simulated muscle activations (*a*) and nondimensionali​se​d fibre lengths (l*) of uniarticular hip muscles with activation >50% of maximum (*a*=1) from dynamic simulations with full tissue properties of the exemplar STS and STW trials. Some other muscles not shown also had activation >50% of maximum, including ITCaa in STS. For STW, the stance leg was simulated. Nondimensionali​se​d fibre lengths are colour coded according to where on the active force–length curve fibres would be operating: steep ascending limb, shallow ascending limb, plateau and descending limb [divisions approximately correspond to Arnold and Delp (2011)]. (A) STS and (B) STW events and phases are denoted, where heel-off is represented by an arrow and the end of the ascending phase is represented by a dashed line. Muscle abbreviations are defined in Table 1.

**Fig. 7.**
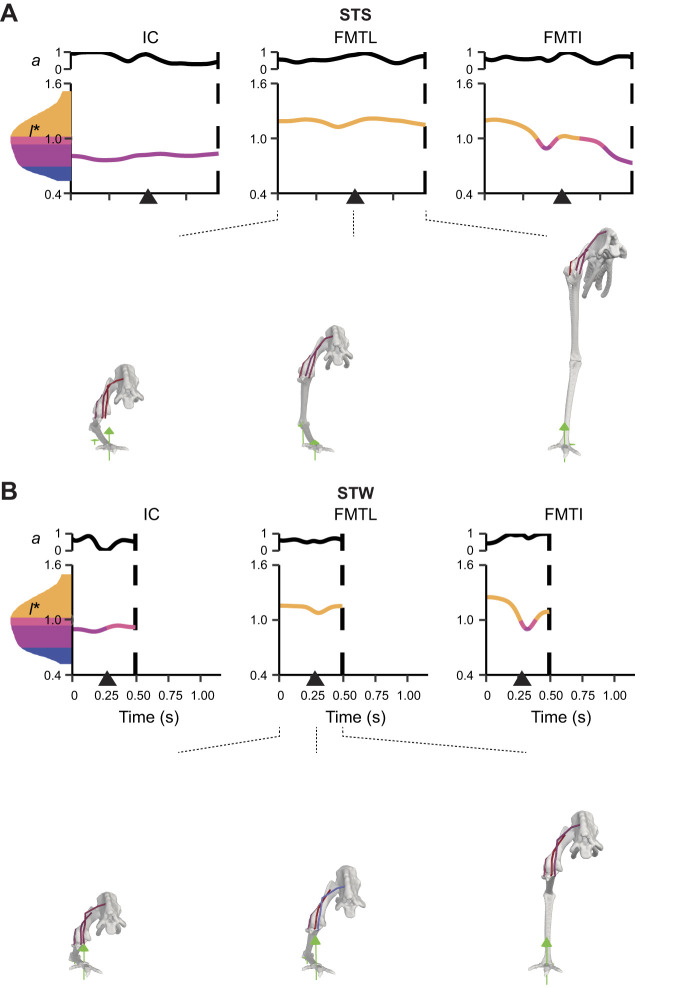
Simulated *a* and l* of biarticular muscles crossing the hip and knee and uniarticular knee muscles. Muscles with activation >50% of maximum (*a*=1) from dynamic simulations with full tissue properties of the exemplar (A) STS and (B) STW trials are shown. Some muscles not shown also had activation >50% of maximum, including ILPR in STS and FMTM in STW. See Fig. 6 for further details.

**Fig. 8.**
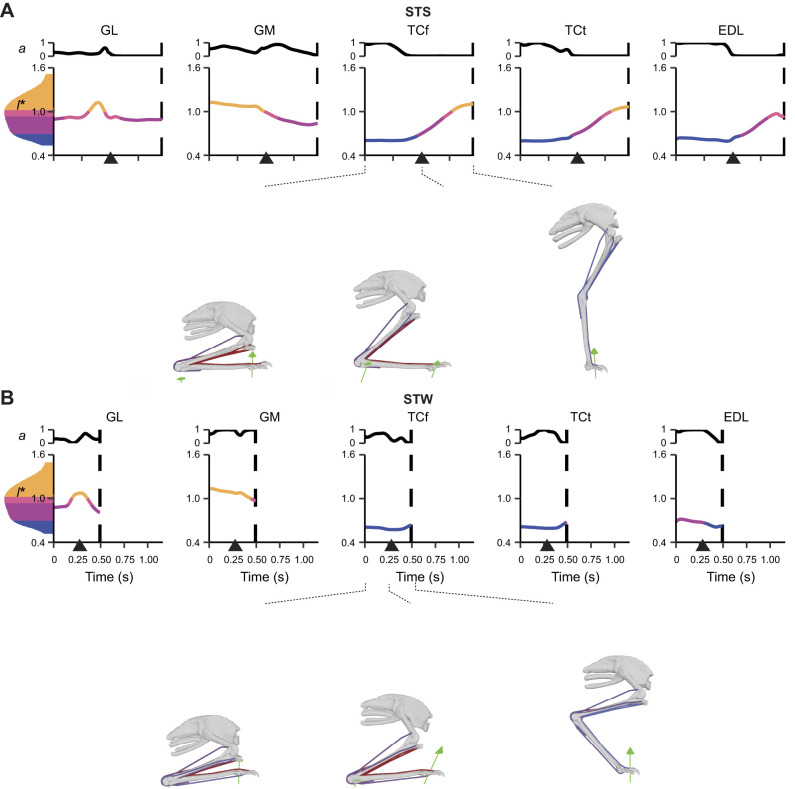
Simulated *a* and l* of biarticular muscles crossing the knee and ankle, and muscles crossing the tarsometatarsophalangeal (TMP) joint. Muscles with activation >50% of maximum (*a*=1) from dynamic simulations with full tissue properties of the exemplar (A) STS and (B) STW trials are shown. Some muscles not shown also had activation >50% of maximum, including FP3 in STS. See Fig. 6 for further details.

Simulation results were broadly similar across the different simulation frameworks (Figs 6–8; Figs S3–S5). Consistent with the joint moment results, simulations estimated that most muscle activations and forces peaked at around heel-off, with a few muscles remaining active through the end of the movements. We focus here on results of the dynamic simulations with full tissue properties and present additional simulation results and sensitivity analyses later. All simulation results are provided in Dataset 1.

#### Near-maximal activations in major extensor muscles

In dynamic simulations of both STS and STW exemplar trials, 12 of 37 muscles reached activation that was >50% of maximum (*a*=1), including major hip, knee and ankle extensors (Figs 6–8). Notably, FMTI and GM remained highly activated throughout the movements, reaching near-maximal activations. Some short-fibred hip muscles whose major functions were non-sagittal also showed near-maximal activations, including ITCr, OMII and OMIP (see Table 1 for full names of muscles analysed).

We also observed co-activations in antagonist (pedal dorsiflexor) muscles such as TCf, TCt and EDL, and medial (e.g. ITCr and ITCaa) and lateral (e.g. OMII and OMIP) rotators. Simulations predicted most other muscles to have lower activations (<50% of maximum). In general, STW exhibited greater or similar peak muscle activations than STS transition when comparing the two exemplar trials, except for a few muscles, such as ITCaa – a hip muscle mainly contributing to non-sagittal motions. Overall, the results demonstrated greater activations in major extensor muscles and specific hip muscles, with STW generally exhibiting greater or similar activations compared with STS.

#### Large fibre length changes

STS and STW dynamic simulations showed moderate to large changes in muscle fibre lengths, with 28.3±14.6% and 18.8±13.3% changes, relative to *l*_o_ in the STS and STW exemplar trials, respectively (Figs 6–8). Distal hindlimb muscles, with a 32.5±9.61% change in fibre lengths during STS, overall underwent larger length changes than proximal muscles (i.e. muscles crossing the hip and/or knee joints) with an average of 25.8±16.6% change in fibre lengths during STS. One exception was FCLA – a proximal muscle crossing the hip joint, which had 65.4% change in its muscle fibre length during the transition. Most of the muscle fibres operated within the range of 0.5–1.5 *l*_o_, except for OMII, which operated below 0.5 *l*_o_ in the STW dynamic simulation.

We also observed unique patterns of lengthening and shortening across muscle groups in the simulation. Some muscles were at extremely long fascicle lengths early in the transition (∼1.5 *l*_o_), such as PIFML, FMTI and GM, and were actively shortening early in the transition or throughout the movement. Other muscles were at their minimum lengths, such as TCf and TCt (∼0.5 *l*_o_). Additionally, some muscles such as IC remained at lengths closer to optimal for force generation. These patterns showed a diversity of behaviours for emu hindlimb muscles during STS and STW, suggesting a diversity of functional roles.

#### Substantial forces in ankle extensor muscles

Consistent with the muscle activation patterns, many muscles generated large forces, approaching or exceeding body weight, especially in the ankle extensors (muscle force results are provided in Dataset 1). GM was distinct, reaching a maximum force of 4.3 BW in STS and 5.5 BW in STW, with FL and GL muscle forces also exceeding 1.5 BW in STS and 2.5 BW in STW. Other major extensor muscles around the hip and/or knee joints, including IC and FMTI, reached maximum forces of more than 1 BW. AMB had strikingly high activations (∼1.0) during STW but only generated small peak forces (∼0.44 BW). EDL, which acted as both digit and ankle dorsiflexor, was co-activated and developed a maximum force 1.7 BW in STS and 1.5 BW in STW. Intriguingly, some muscles contributing to non-sagittal motions, including ITCr, OMII and OMIP, also generated moderate (∼1.0 BW) to large forces, with OMIP peaking at around 4.5 BW. From the start of the transition until the ascending phase, STW necessitated larger forces in most muscles compared with STS, with a few exceptions, including ILPR, ITCaa, OMII, TCt, EDL and FP3. Overall, the results showed that ankle extensor muscles generated substantial forces, often exceeding body weight, suggesting large demands during STS and STW.

### Sensitivity analyses

The simulated muscle activations, fibre length changes and force patterns showed broad similarities between the static (assuming rigid tendons) and dynamic (incorporating full tissue properties) simulations, with some quantitative differences (Fig. S3–S5). In both STS and STW static simulations, 21 out of 37 muscles achieved activations exceeding 50% of maximum. Similarly to the dynamic simulations, STS and STW static simulations demonstrated moderate to large changes in muscle fibre lengths, averaging 28.9±16.4% and 21.3±15.8% relative to *l*_o_ in the exemplar trials. The distal limb muscles crossing the ankle and/or TMP joints were particularly notable, exhibiting an average 33.8±10.4% change in fibre lengths during STS. In static simulations, muscle forces were generally comparable to or greater than those in dynamic simulations across most muscles, but smaller in some major distal limb muscles such as GM, FL, TCf and TCt (discussed below). Additional dynamic simulations with rigid tendons (provided in Dataset 1) showed similar muscle activation patterns and forces as static simulations, but slightly reduced reserve actuator requirements. This suggested minimal differences in their outcomes between the static simulation in OpenSim and dynamic simulation with rigid tendons in OpenSim Moco. These findings together indicated that tendons had various roles during STS and STW, in reducing muscle activations, excessive fibre length changes and muscle forces.

Static simulation results were influenced by ±5% *l*_s_ changes for numerous muscles, particularly when the ratio of muscle fibre length to tendon length was smaller (Figs S3–S5, dashed and dotted lines). Hip and knee muscles showed lower sensitivity to *l*_s_ changes, although activations and forces early in the transition were impacted. Patterns for l* for specific muscles, such as PIFML and FMTI, were moderately shifted closer to their optimal length limit (for shorter tendons) or moved toward/below the optimal length (for longer tendons). In contrast, muscles acting about the ankle and digits displayed substantial changes in activation, length patterns, and forces. For instance, in the STS simulation, GM notably generated greater force (4.5 BW, compared with 4.1 BW in the nominal simulation) with smaller fibre length change when *l*_s_ was increased by 5%, and smaller force (3.8 BW) with larger fibre length change when *l*_s_ was decreased by 5%. These changes were reflected in l* in the same ways as for proximal muscles, where decreased *l*_s_ values led to longer fibre lengths.

**Fig. 9.**
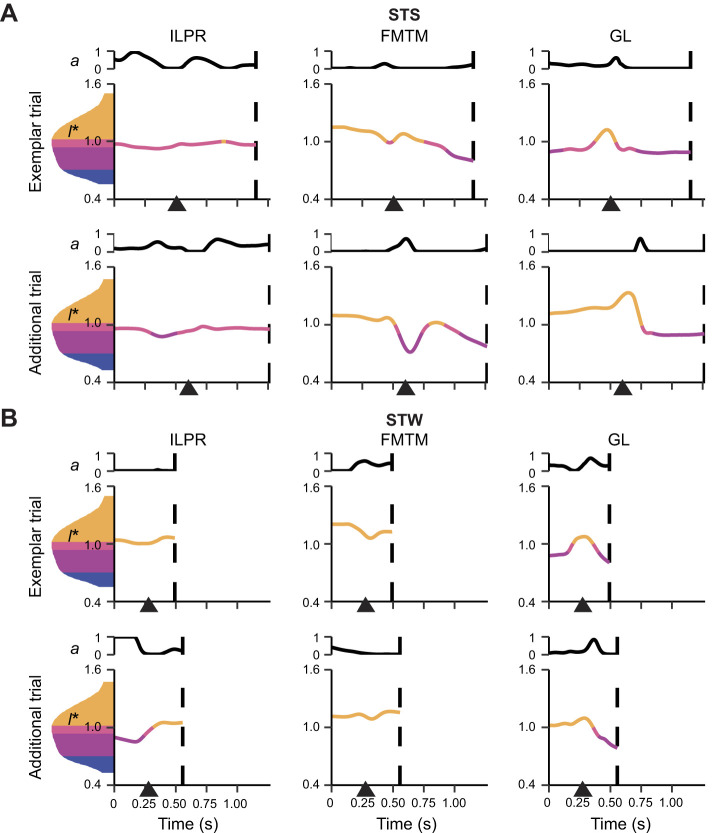
Simulated *a* and l* of the representative muscles during STS and STW in the exemplar and additional trials. Results from the nominal static simulations are shown for (A) STS and (B) STW. See Fig. S3 for further details.

With a few notable differences, the simulations of the two additional trials (one for each behaviour) generated similar results (Fig. 9). Activation levels were similar or lower in the additional STS trial. For example, GL was activated at the onset of STS in the exemplar trial but not until after heel-off in the additional trial. Activation levels remained broadly similar in the STW trials, except for ILPR, which was not activated in the exemplar trial but reached near-maximal activation in the additional trial. Some muscles showed shifted peak activations in early or late transition, such as FMTM in STW simulations. In terms of fibre lengths, FMTM in the additional STW simulation lengthened during the transition, which was opposite to results in the exemplar trial. GL started at the ascending limb of its force–length curve in both STS and STW exemplar trials but at the descending limb in the additional trials. In general, while some quantitative results varied between the trials, qualitative patterns were broadly similar, such as activations and forces peaking at around heel-off and substantial fibre length changes.

### Reserve actuators

With a few exceptions, reserve actuator values in the nominal static (assuming rigid tendons) and (incorporating full tissue properties) dynamic simulations remained small in comparison to the inverse dynamics joint moments (typically <1 Nm or ≤10% of average or peak inverse dynamics moments; provided in Dataset 1). The main exception occurred at the ankle joint, where large reserve actuator moments were required in flexion/extension DOF (i.e. a maximum of 5.3 Nm in STS and 2.8 Nm in STW in dynamic simulations) and abduction/adduction and long-axis rotation moments were mainly attributed to reserve actuators. Generally, altering *l*_s_ by ±5% increased the demand for reserve actuator moments, particularly in distal limb joints. The high sensitivity in distal limb muscles to *l*_s_ changes indicated that reserve actuators were directly compensating for the reduced muscle capacity to generate the moments required for STS and STW. Compared with static simulations, dynamic simulations incorporating full tissue properties reduced reserve actuator moments at distal limb joints in both movements (e.g. the maximum ankle flexion/extension reserve actuator accounted for 25% in static simulation and 15% in dynamic simulation in STS). It was notable that although STW required larger ankle flexion/extension joint moments than STS transition (Fig. 5), reserve actuator moments were smaller compared with net joint moments (i.e. a maximum of 15% in STS and 7.2% in STW), suggesting a more effective use of muscles contributing to ankle extension. However, STW required larger percentages of reserve actuator moments around the hip and knee joints.

## DISCUSSION

Our study presents the first dataset of hindlimb kinematics and kinetics as well as simulated muscle dynamics during emu sit-to-stand and sit-to-walk transitions; or for any bird. Our findings overall support all four hypotheses concerning emu hindlimb muscle activations, fibre length changes, passive tissue roles, and differences in muscular demands between STS and STW.

Supporting hypothesis 1, our simulations revealed substantial activations and forces in many extensor muscles as well as key muscles contributing to non-sagittal movements during both STS and STW (Figs 6–8). Major hip, knee and ankle extensors such as PIFML, FMTI, GL and GM had large activations and forces. Although emus primarily required flexor moments around the knee joints during the movements, the knee extensor muscles were likely activated to counter the knee flexion moments generated by ankle extensors. Moreover, we found that muscles transitioning from initially sub-optimal fibre lengths (e.g. GM) had higher forces than those operating closer to optimal length values (e.g. IC), suggesting the use of passive forces. Co-contractions of extensor and dorsiflexor muscles and medial and lateral rotator muscles were also observed, particularly early in the transition phase, probably serving to stabilise the hip and pedal joints during standing up.

Hypothesis 2 postulated that emu hindlimb muscles would operate near their functional limits. Unlike the more isometric muscle patterns observed in forward locomotion of birds (e.g. Bishop et al., 2021c; Daley and Biewener, 2003; Roberts et al., 1997), our nominal static simulation results showed extensive fibre length changes during standing up, closely approaching their functional limits (0.5–1.5 *l*_o_), especially in distal muscles (Figs 6–8). Some muscles such as GL and FP3 were further away from this limit but exhibited more sensitivity to changes in *l*_s_ and simulation frameworks. In our model building approach, we systematically adjusted *l*_s_ and *l*_o_ in order to keep muscle fibres within a reasonable operating range. While this process could have biased the analysis towards (or even away from) extreme muscle lengths, our modifications were cautious, ensuring that muscles avoided excessive lengthening or shortening. Thus, we contend that the approach was appropriate (discussed below).

Hypothesis 3 proposed the necessity of passive support mechanisms (namely tendons), especially in the distal hindlimbs, for executing STS and STW tasks. Modifying *l*_s_ by ±5% notably increased muscle activations for most muscles, with many reaching near-maximum levels (∼1.0) (Figs S3–S5). This modification also amplified the demand for reserve actuator moments, especially in the distal hindlimbs, where muscle fibres approached their functional limits. This outcome was not surprising, considering that muscles operating beyond their optimal length ranges had greater difficulties in generating adequate forces (Zajac, 1989; Millard et al., 2013). Muscle activations and fibre length changes were generally smaller in dynamic simulations (incorporating full tissue properties) than in static simulations (assuming rigid tendons), especially for distal muscles (Figs 6–8; Figs S3–S5). Furthermore, dynamic simulations notably reduced the necessity for reserve actuators at distal limb joints, highlighting the crucial role of passive MTU tissues such as tendons and aponeuroses during standing up. Interestingly, allowing for compliant tendons resulted in smaller forces for most of the muscles, but larger forces for some ankle extensors (Figs 6–8; Figs S3–S5). This discrepancy may be attributed to the fact that the increased force generation capacity of some muscles (e.g. GM and FL) may reduce the muscle force requirements of other muscles and/or level of co-contraction acting around the same DOF (e.g. GL, FP3 and FPP3), while the total required joint moments remained constant. Subsequent investigations that quantify muscle work could contribute to a clearer understanding of these dynamics. Overall, our results indicated the important roles of tendons in preventing large muscle activations and fibre length changes.

Hypothesis 4 proposed that STW would involve greater demands on muscle coordination than STS in emus, predominantly for extensor muscles and non-sagittal muscles. We found that STW involved greater non-sagittal motions at the hip and knee than in STS (Fig. 3; Table S1). Consequently, in emus, muscle coordination in STW may be more demanding than that in STS in order to meet the demands of non-sagittal joint moments. Simulation results support this notion, demonstrating that STW generally required greater activations and muscle forces than STS (Figs 6–8). STW also required reserve actuator moments that were greater for the hip and knee joints compared with STS, but smaller for the ankle extension/dorsiflexion DOF. Our results suggest that compensatory strategies may be used in STW to prevent large ankle extensor activations with elevated hip and knee muscle activations (discussed below).

The movement patterns of emu STS and STW exhibited similarities and distinctions when compared with humans. Emus demonstrated a sequential joint excursion from proximal to distal during the transition like humans do (Pandy et al., 1995); an observation also seen in greyhounds (Ellis et al., 2018). Both emu movements involved generating forward momentum, positioning the COM behind the toes at heel-off, resembling the ‘momentum transfer’ or ‘forward momentum’ strategy observed in humans (Hughes, 1996; Norman-Gerum and McPhee, 2020; Perera et al., 2023). However, it is important to note the differences between species: emus exhibited a small caudal GRF at the onset of the movement (Fig. 4); a pattern not typically observed in humans (during STS, STW and deep squat-to-stand movements) or greyhounds (Ellis et al., 2018; Hoogenboom et al., 2023; Kralj et al., 1990; Perera et al., 2023). This caudal GRF induced a slight backward shift of the body at the beginning of the movement, which allowed for greater displacement during the forward momentum phase, ultimately increasing the applied impulse and enhancing vertical momentum during STS and STW. Additionally, compared with STS, STW demands greater stability in humans, often with a longer forward (or downward pitch) momentum phase sacrificing speed or efficiency (Kerr et al., 2004). However, emus had a relatively small initial trunk flexion movement and displayed a faster forward momentum phase in STW than in STS (Fig. 3; Table S1). Emus also seemed to have a relatively smaller braking impulse compared with humans during the transition (e.g. Magnan et al., 1996; Perera et al., 2023) when performing the STW task (Fig. 4; Table S1). The braking impulse reduces forward momentum and presumably allows a focus on stability and postural control when standing up (Perera et al., 2023). These differences suggest that emus transition from sitting to walking while maintaining stability, possibly because of their cranially positioned COM and use of passive support mechanisms. This speculation remains untested but aligns with hypotheses on birds' energy-efficient passive stabilisation methods (Abourachid et al., 2023). Further support for this idea comes from the smaller medio-lateral COP (Table S1) – which is regarded as a balance indicator (Thompson et al., 2017) – that is observed in STW relative to STS.

While direct measurements of emu hindlimb muscle forces and electromyographic data are unavailable, our simulation results suggested that emus had many muscle activation patterns that were similar to those in humans, with a few noticeable differences. We did not observe an obvious proximal-to-distal muscle activation sequence (despite proximal-to-distal joint motions) like that observed in human STS (Pandy et al., 1995), and a few muscles (e.g. GM) remained continuously active throughout the transition. Similar results were also found in previous research on greyhound STS (Ellis et al., 2018). Across all simulations, we observed co-contractions of antagonist muscles, which are likely to be crucial, not only for stability, but also for redistribution of joint moments (e.g. Actis et al., 2018; Khemlani et al., 1999; Roebroeck et al., 1994; Savelberg et al., 2007; Shia et al., 2018). In contrast to humans, emus required a relatively small net extensor moment around the knee joint (Fig. 5), probably as a result of their more horizontally oriented trunk and cranially positioned COM. The latter two traits, however, would potentially increase the load on ankle extensors. This redistribution of moments becomes particularly evident in STW, which is characterised by increased net joint moments around the hip and ankle joints, with increased trunk flexion and a more forward position of the COP (Fig. 5). The trade-offs between fibre lengths and moment-generating capacity pose additional challenges for ankle extensors, suggesting that the ankle extensor capacity represents a key biomechanical constraint in cursorial species during standing up, as well as potentially in running (Hutchinson, 2004).

Our study identified three possible movement strategies or anatomical adaptations that emus use to navigate biomechanical challenges during standing up: relying on key pelvic limb muscles beyond just the extensors, using substantial movement in non-sagittal planes and leveraging the elastic properties of their tendons. Emus coordinated substantial non-sagittal motions of the hips and knees to execute STS and STW, which were similar to those observed in various maneuvers by Kambic et al. (2014, 2017). These findings underscore the importance of analysing 3D joint coordination in these movements across bird species. We initially presumed that emus would rely on hip abductors and medial rotators to counteract the adduction forces exerted by the GRF due to the quasi-parasagittal gait typical of birds (Allen et al., 2021; Gatesy, 1994, 1999; Hutchinson and Gatesy, 2000), as well as findings from simulation studies of muscle forces and bone strains in stance (Goetz et al., 2008; Rankin et al., 2016; Wei and Zhang, 2023). However, during STS and STW, emus required net moments to counter hip abduction (Fig. 5) and exhibited co-contraction of medial and lateral rotators, particularly around heel-off, indicating the critical functional role of those non-sagittal muscles in their standing-up movements. The tendons' roles during STS and STW were mainly in reducing large fibre length changes and excessive muscle forces, particularly in distal muscles – an observation consistent with previous research on greyhounds (Ellis et al., 2018). Walking and running simulations of other birds have also highlighted the necessity of tendons for passive support in non-sagittal motions and distal limb joints (Bishop et al., 2021c; Daley and Biewener, 2003; Rankin et al., 2016; Roberts et al., 1997). However, the efficacy of tendons in elastic energy storage and return during STS and STW might be limited compared with their roles in running (e.g. Maloiy et al., 1979; Roberts et al., 1998; Rubenson et al., 2011) or jumping (e.g. Bishop et al., 2021a; Henry et al., 2005; Konow and Roberts, 2015). The challenge is that muscles need to maintain quasi-isometric conditions to effectively recover strain energy when tendons recoil (Biewener, 1998). Tendon elastic energy storage and return depends on rapid loading and unloading, which differs from the demands of standing up. This suggests various roles of tendons in different behaviours, offering insights into how musculotendon anatomy may need to adapt for specific behaviours and revealing trade-offs for some muscles across different behaviours (e.g. locomotor vs non-locomotor behaviours).

This study rested on two pivotal assumptions: bilateral symmetry and the roles of tendons. Although we assumed bilateral symmetry during the ‘forward momentum’ and ‘ascending’ phases, asymmetrical movements or forces between contralateral limbs across different planes were likely, an observation also seen in human STS and STW studies (e.g. Boukadida et al., 2015; Caruthers et al., 2016; Dolecka et al., 2015; Lundin et al., 1995). This asymmetry might serve as a compensatory mechanism during forward acceleration of the COM (van der Kruk et al., 2021) or for quicker balance recovery (Blaszczyk et al., 2000). In addition, simplifications in our model might limit a comprehensive understanding of the roles of tendons. First, our model did not include other passive tissues, such as ligaments, which are considered to be highly specialised for energy savings in large ratites (e.g. Badri-Spröwitz et al., 2022; Schaller, 2008; Schaller et al., 2009). Second, the Hill-type model's application might vary across different species and behaviours. For example, the stretch-shortening effect of muscles (Anderson and Pandy, 1993; Bobbert and Casius, 2005; Daley and Biewener, 2003) could also play a role during standing up in some muscles such as GL, which underwent active lengthening and then shortening. Third, tuning *l*_s_ to maintain muscle fibre lengths within a feasible range currently lacks a standardised approach (e.g. Redl et al., 2007; Scovil and Ronsky, 2006). Owing to the logistic difficulties in adjusting muscle parameters consistently across different simulation frameworks, adjustment of *l*_s_ values were based on a rigid tendon assumption, which did not consider muscle–tendon interactions, resulting in some fibres operating at less than 0.5 *l*_o_ even in dynamic simulation with full tissue properties (e.g. OMII), but compliant tendons still did reduce fibre length changes. The ‘Muscle Redundancy Solver’ package offers a relatively robust and computationally efficient way of tuning muscle parameters using the direct collocation optimal control method (De Groote et al., 2016). This approach, as shown for tinamou birds by Bishop et al. (2021c) can account for modelling or measurement errors that might otherwise impede a musculoskeletal model's ability to execute a recorded behaviour. However, it also involves some subjectivity, as it relies on the estimation of measurement errors to determine the range of adjustment for each parameter.

The sample size of the study, calculation of joint angles and single limb GRFs, and the modelling approach in our study come with limitations that should also be acknowledged. First, the study sample size was limited by the challenges of handling and training the birds and ethical considerations. From 39 successfully recorded STS and STW trials, we applied a set of necessary inclusion and exclusion criteria, which led to a final dataset of 3 STS and 9 STW trials (Supplementary Materials and Methods). The discrepancy between STS and STW trials was not unexpected, as STW is a more common daily activity where the end goal is walking. Second, joint kinematics computed from skin markers can be substantially influenced by factors such as calibration accuracy (Chiari et al., 2005), skin movement (Leardini et al., 2005) and marker placement errors (Della Croce et al., 2005). The criteria for measurement accuracy also were derived originally from human studies (Hicks et al., 2015). Despite our best efforts to account for these challenges, we still observed large variations in knee and ankle rotation angles, potentially attributed to small errors in the placement of tibiotarsus markers (Fig. S2). Third, the partitioning of GRFs based on the assumption of bilateral symmetry may lead to an underestimation of actual GRFs, particularly notable for the stance limb during STW. Additionally, imprecise COP positions could also influence inverse dynamics moments. While a more comprehensive procedure would improve accuracy (e.g. Rubenson et al., 2007), it would require new data collection and reanalysis of newly generated simulations. Alternative methods, such as tracking simulations that partly account for error in experimental data, could mitigate inconsistencies between model kinematics and kinetics (e.g. Bishop et al., 2021c). Finally, our simulations required the use of reserve actuators, which can be partly attributed to the unmodelled passive joint support mechanisms mentioned earlier. Additionally, in demanding movements, the strength of the modelled muscles may not be sufficient to generate the required joint torques, as shown in previous studies (e.g. Hof et al., 2002; Hutchinson, 2004; Rankin et al., 2016). Despite these limitations, the simulations only had small reserve torques. Incorporating upper body movement including head, neck and wings into our model may yield additional insights into STS and STW, analogous to situations where humans use arm movements to aid in standing up (Davidson et al., 2013; Dolecka et al., 2015; Komaris et al., 2016; Mazzà et al., 2004). For instance, the initial forward motion of the neck and head during these transitions might contribute slightly to generating forward momentum (Movies 2 and 3). However, because of the relatively small mass of the neck and head in emus, their impact may be less significant than initially expected. Wing movement could potentially play a more substantial role in stabilisation, particularly during STS. However, our observations indicate minimal wing movements in emus during these transitions (Movies 2 and 3). Instead, emus appear to rely predominantly on trunk movements, suggesting unique constraints and strategies during their standing-up movements.

### Conclusions

As the first investigation on sit-to-stand and sit-to-walk transitions in an avian species (or even among very few for non-humans in general), this study unravels joint mechanics, constraints and compensatory strategies used by emus during these movements. Emus demonstrate large muscle activations, substantial muscle force requirements and fibre length changes, particularly in their distal hindlimbs, with ankle extensors acting as a key biomechanical limit during standing up. To mitigate trade-offs between muscle capacity and fibre lengths, emus use non-sagittal muscle actions and tendon length changes, which is more evident in transitioning from sitting to walking. The current study lays a groundwork for broader investigations across diverse species and larger sample sizes. Understanding the foundational biomechanics of sit-to-stand and sit-to-walk transitions in terrestrial animals holds promise for insights into morphofunctional specialisations, body size influences, evolutionary studies, applications in robotics and advancements in animal welfare.

## Supplementary Material

10.1242/jexbio.247519_sup1 Supplementary information

Dataset 1.
