# Supplementary material for: Hindlimb kinematics, kinetics and muscle dynamics during sit-to-stand and sit-to-walk transitions in emus (Dromaius novaehollandiae)
Source: J Exp Biol. 2024 Dec 2;227(24):jeb247519. doi: 10.1242/jeb.247519 (PMC11708823; doi:10.1242/jeb.247519)
Supplement: Supplementary information [file jexbio-227-247519-s1.pdf]

Supplementary Materials and Methods

Marker set definitions for emu trials

The following ‘Marker set’ lists the markers shown in **Fig. 2A**.

Segment	Marker set	Definitions
Body	TR1-TR3	Three markers (placed along the spine) used to define the plane of the pelvis.
Femur	FC1-FC3	Three markers used to define the femur orientation.
	HJC	Marker locating the hip joint centre.
Tibiotarsus	TBC1-TBC3	Three markers used to define the tibiotarsus.
	KJC	Marker locating the knee joint centre.
Tarsometatarsus	TSC1-TSC3	Three markers used to define the tarsometatarsus.
	AJC	Marker locating the ankle joint centre.
Digits	T1-T3	Three markers used to define the phalanges segment (T1: digit IV, T2: digit III, T3: digit II).
	TMPJC	Marker locating the tarsometatarsophalangeal joint centre.

Trial inclusion criteria

We performed approximately 30 trials for each of the three individuals, resulting in 39 successfully recorded sit-to-stand/walk trials: 12 from Emu O, 15 from Emu R, and 12 from Emu G. To refine this dataset for analysis, we first excluded trials where emus initiated movement from a semi-squatting rather than a fully recumbent posture. As a result, all 12 trials of Emu G and a subset of 7 trials from Emu O and 5 from Emu R were excluded, leaving us with 5 trials from Emu O and 10 from Emu R that were potentially viable for analysis.

Given the small dataset, we applied distinct inclusion/exclusion criteria for kinematic and kinetic data separately. For kinematic analysis, we omitted trials with markers showing non-rigidity or absence, leading to unnatural movements. Visual confirmation via inverse kinematics and video examination resulted in retaining 5 sit-to-walk (STW) trials from Emu R (including 3 stance leg trials and 2 swing leg trials), 1 STW trial from Emu O (swing leg), and 3 sit-to-stand (STS) trials from Emu R for analysing kinematic outcomes such as angles and velocities.

Regarding kinetic data analysis, specific inclusion criteria were implemented: both limbs on the force plate and a peak vertical ground reaction force (GRF) within 0.9 to 3.5 times the body weight range, ensuring minimal interference from other force plates. This process yielded 5 STW trials from Emu R (comprising 4 stance leg trials and 1 swing leg trial), 3 STW trials from Emu O (including 1 stance leg trial and 2 swing leg trials), and 3 STS trials from Emu R for analysing kinetics, such as GRFs and centre of pressure.

Ultimately, we chose two representative STS/STW trials and two additional trials from Emu R based on qualitative assessments. These selections were made considering data within observed kinematic and kinetic ranges, focusing on near-bilateral symmetry, characterised by minimal medio-lateral GRF and medio-lateral centre of pressure.

Journal of Experimental Biology • Supplementary information

Scaling of model to fit experimental subject

As per the Methods, the model was scaled in OpenSim to match the subject's dimensions. The scaling factors of the model to two experimental subjects (EMU R, 28.5 kg and EMU O, 27.4 kg) were based on ratios of bone segment lengths.

EMU R:

Body segment: 0.832 * Model animal size

Femur segment: 187 mm / 219 mm * Model animal size

Tibiotarsus segment: 327 mm / 407 mm * Model animal size

Tarsometatarsus segment: 323 mm / 380 mm * Model animal size

Digits segment: 50 mm / 61 mm * Model animal size

EMU O:

Body segment: 0.822 * Model animal size

Femur segment: 200 mm / 219 mm * Model animal size

Tibiotarsus segment: 340 mm / 407 mm * Model animal size

Tarsometatarsus segment: 299 mm / 380 mm * Model animal size

Digits segment: 46 mm / 61 mm * Model animal size

GRF partitioning

We modelled the hindfoot as comprising two rigid segments: the 'tarsometatarsus' and the 'digits', whereby the tarsometatarsus was composed of the fused distal tarsals and metatarsals II, III and IV, and the digit was composed of the phalanges of the three digits; see below for details on model DOFs. The two segments were connected by the third tarsometatarsophalangeal (TMP) joint coordinate system (see below for details). The GRFs and free moments recorded by the force plates pertained to the foot as a whole, with a

dynamically varying centre of pressure (COP) location. To apply these to the two segments forming the foot in the musculoskeletal model, this necessitated the partitioning of GRFs and free moments appropriately between the tarsometatarsus and digits. To do so, we *a priori* designated the point of application of GRFs and free moments (GRMs) for the two segments as their respective contact point to the ground based on geometry; that is, the COP for each segment remained fixed in location with respect to each segment. The instantaneous positions of the segment contact points to the ground throughout the duration of a given trial were computed using the PointKinematics tool in OpenSim. Then, a custom R script (Dataset 1) was used to partition the recorded GRFs, and the vertical GRM (M_y), between the tarsometatarsus and digit segments as follows (Eqns S1, S2):

$$\left(F_{x,pes}, F_{y,pes}, F_{z,pes}, M_{y,pes}\right) = \left(\begin{array}{c} \left(F_{x,forceplate}, F_{y,forceplate}, F_{z,forceplate}, M_{y,forceplate}\right) \text{ if } d \leq 0 \\ \left((1-d) \cdot F_{x,forceplate}, (1-d) \cdot F_{y,forceplate}, (1-d) \cdot F_{z,forceplate}, (1-d) \cdot M_{y,forceplate}\right) \text{ if } 0 < d < 1 \\ (0, 0, 0, 0) \text{ if } d \geq 1 \end{array}\right)$$

(S1).

$$\left(F_{x,digits}, F_{y,digits}, F_{z,digits}, M_{y,digits}\right) = \left(\begin{array}{c} (0, 0, 0, 0) \text{ if } d \leq 0 \\ \left(d \cdot F_{x,forceplate}, d \cdot F_{y,forceplate}, d \cdot F_{z,forceplate}, d \cdot M_{y,forceplate}\right) \text{ if } 0 < d < 1 \\ \left(F_{x,forceplate}, F_{y,forceplate}, F_{z,forceplate}, M_{y,forceplate}\right) \text{ if } d \geq 1 \end{array}\right)$$

(S2).

where d denotes the relative distance of the COP (recorded by the force plates) with respect to the vector running from the centre of mass of the tarsometatarsus (P1) to the centre of mass of the digit (P2), expressed as a projection via the dot product (Eqn S3):

$$d = \frac{\overline{P_1P_0} \cdot \overline{P_1P_2}}{\left|\overline{P_1P_2}\right|^2}$$

(S3).

Journal of Experimental Biology • Supplementary information

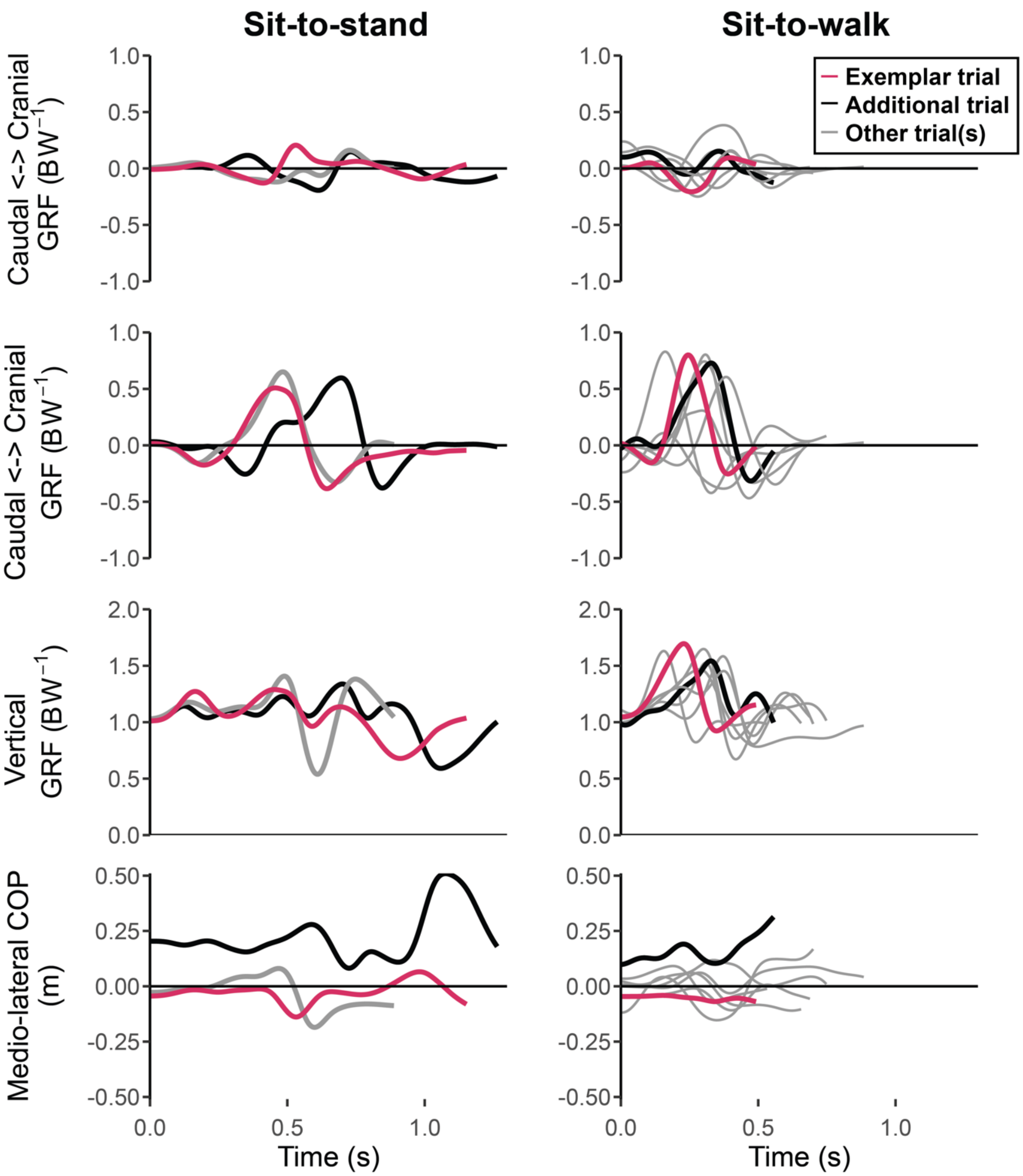

Fig. S1. Total (dimensionless) ground reaction forces and medio-lateral (ML) COP relative to COM during STS in three individual trials from one individual and STW in eight trials from two individuals.

Journal of Experimental Biology • Supplementary information

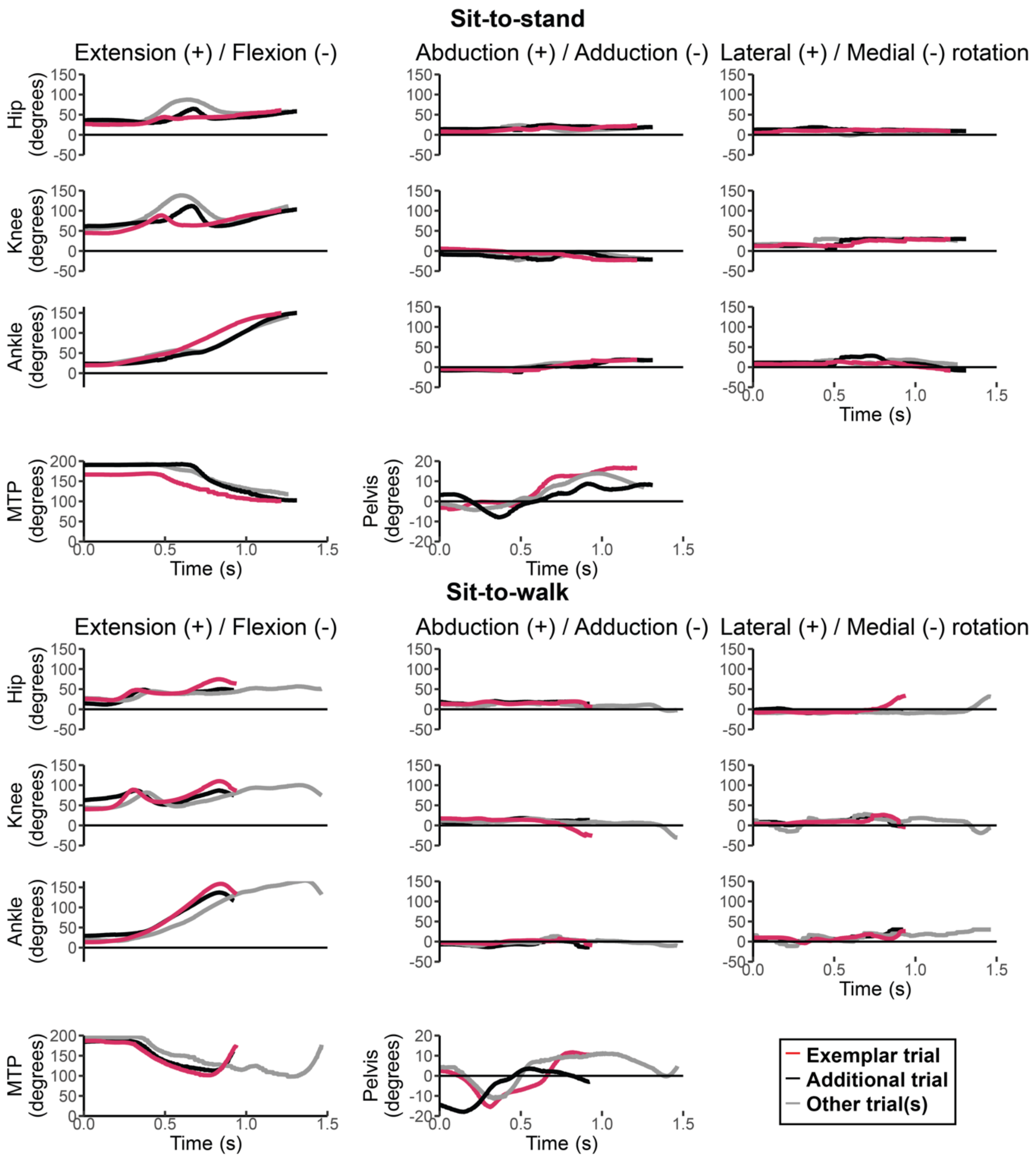

Fig. S2. Body and hindlimb joint angles during STS and STW in three individual trials from one individual. See **Figure 1** for marker/angle definitions.

Journal of Experimental Biology • Supplementary information

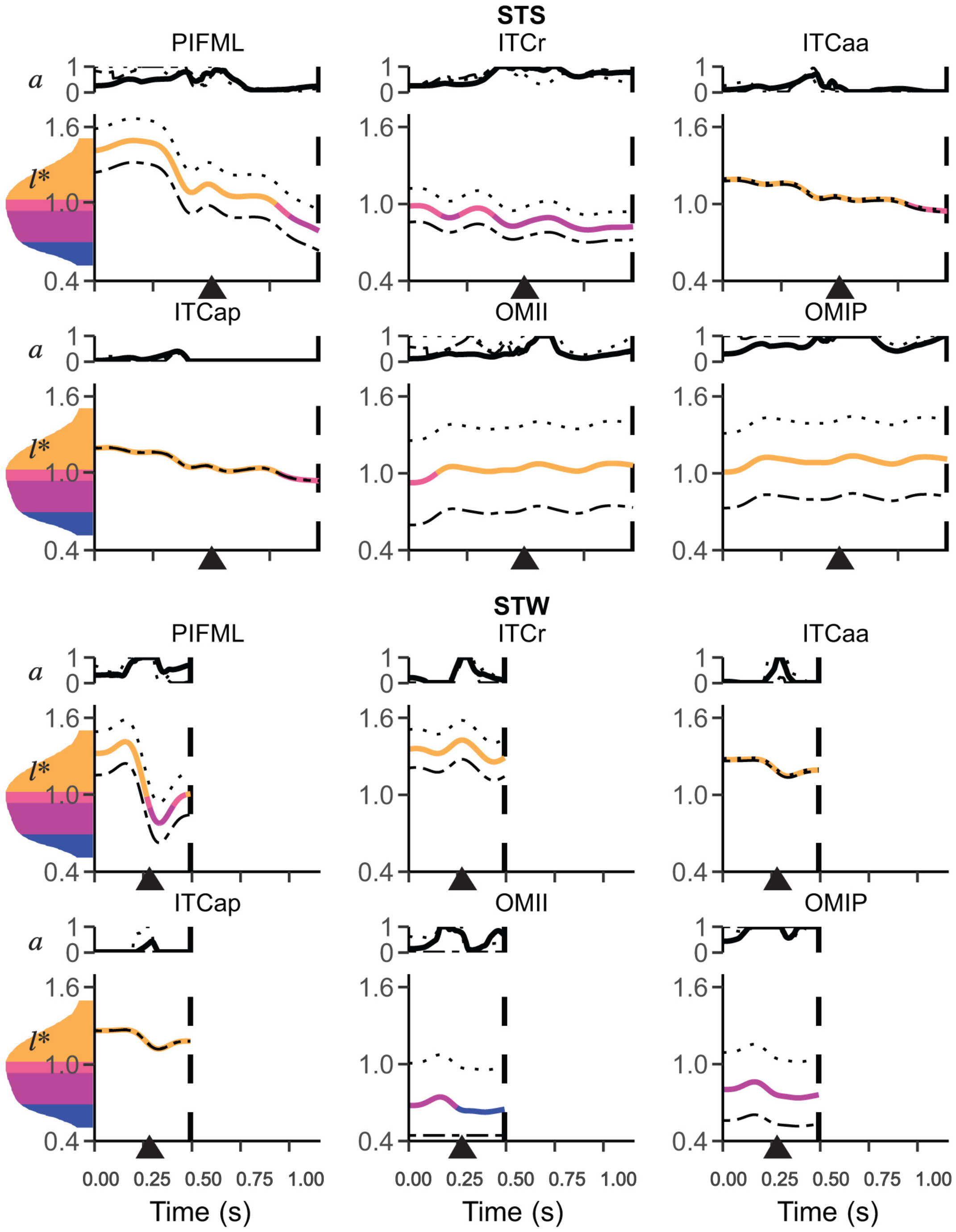

Fig. S3. Simulated muscle activations (*a*) and nondimensionalised fibre lengths (*l) of uniarticular hip muscles with activation >50% of maximum (*a*=1) from nominal static simulations of the exemplar STS and STW trials.** Some other muscles not shown also had activation >50% of maximum, including CFP in STW. For STW, the stance leg was simulated. Results from static simulations (solid line: nominal simulation; dashed line: +5% TSL; dotted line: -5% TSL) are shown. Nondimensionalised fibre lengths are colour coded according to where on the active force-length curve fibres would be operating: steep ascending limb, shallow

Journal of Experimental Biology • Supplementary information

ascending limb, plateau, and descending limb [divisions approximately correspond to (Arnold and Delp, 2011)]. (A) STS and (B) STW events and phases are denoted, where heel-off is represented by an arrow and the end of the ascending phase is represented by a dashed line. Muscle abbreviations are defined in **Table 1**.

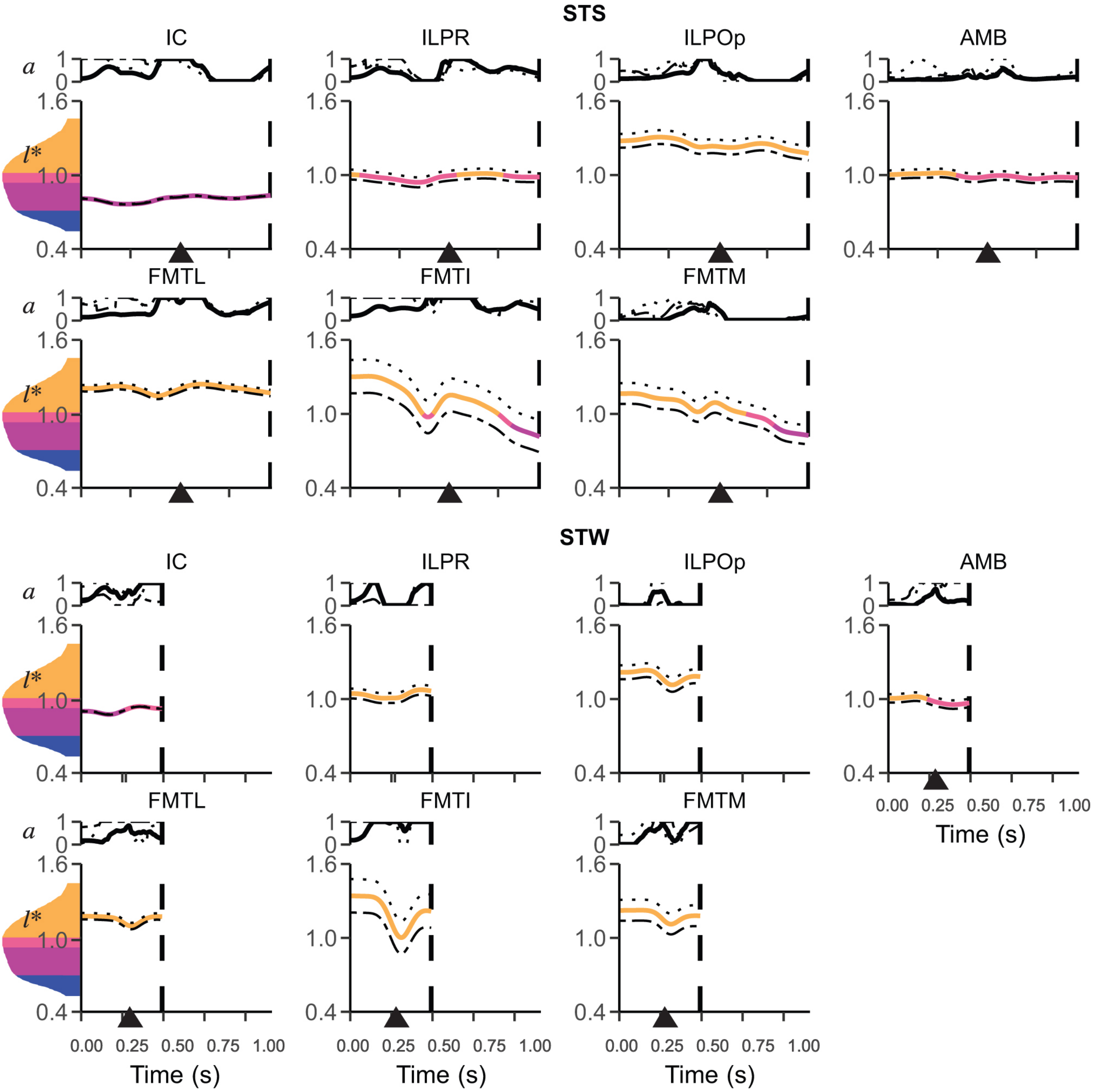

Fig. S4. Simulated *a* and *l of biarticular muscles crossing the hip and knee and uniarticular knee muscles.** Muscles with activation >50% of maximum (*a*=1) from nominal static simulations of the exemplar (A) STS and (B) STW trials are shown. Some muscles not shown also had activation >50% of maximum, including ILPOa in STS and FCM in STW. See **Fig. S3** for further details.

Journal of Experimental Biology • Supplementary information

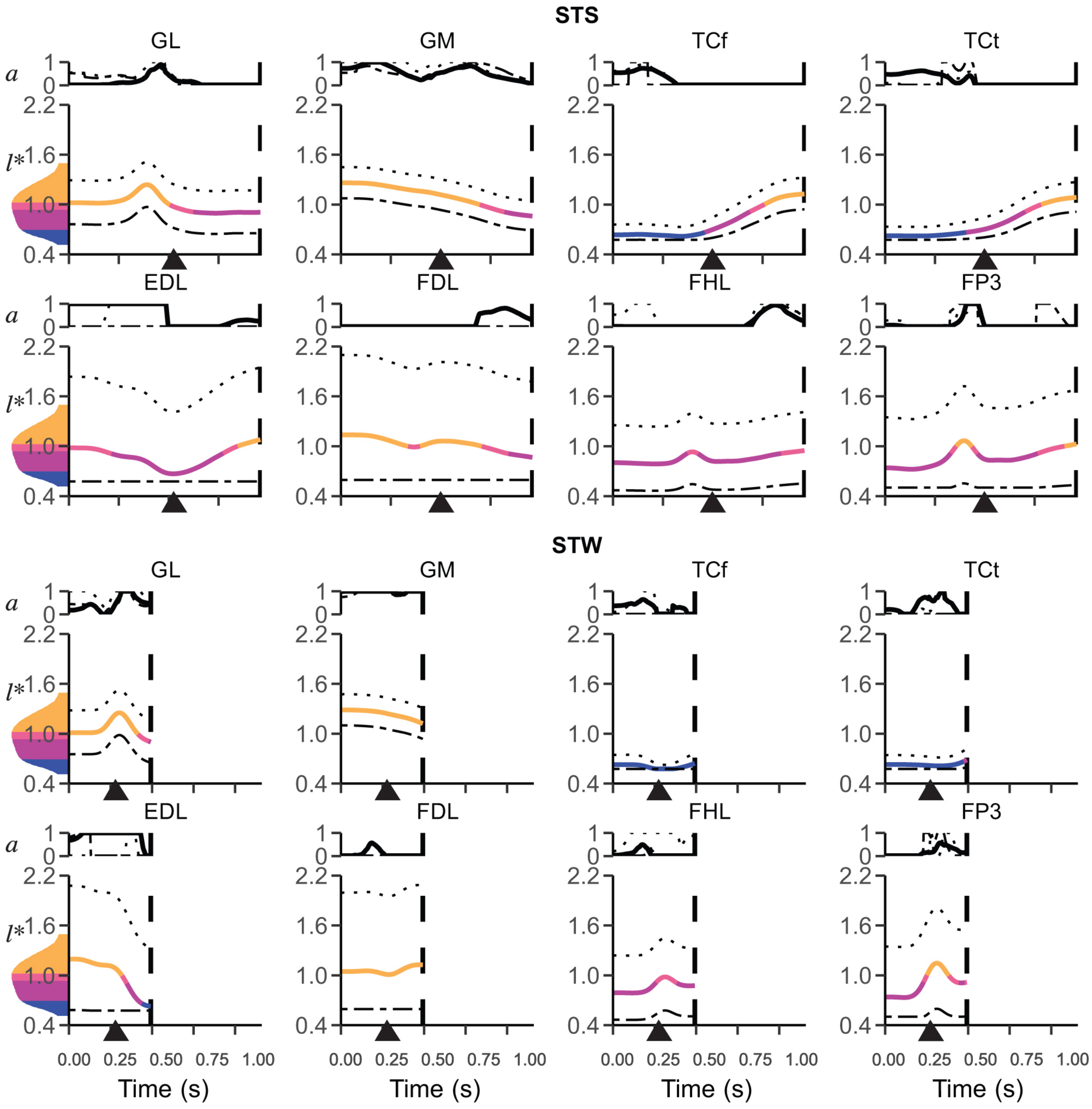

Fig. S5. Simulated *a* and *l of biarticular muscles crossing the knee and ankle, and muscles crossing the TMP joint.** Muscles with activation >50% of maximum (*a*=1) from nominal static simulations of the exemplar (A) STS and (B) STW trials are shown. Some muscles not shown also had activation >50% of maximum, including GI in STW. See **Fig. S3** for further details.

Table S1. Kinematic and kinetic parameters during STS and STW.

Biomechanical parameters		Motion	
		Sit-to-stand	Sit-to-walk
Total time (s)		1.26 ± 0.0481 (1.216)	1.11 ± 0.307 (0.94)
Onset - Heel-off time (s)		0.588 ± 0.0843 (0.500)	0.300 ± 0.0485 (0.272)
Pelvis	Pitch (°)	18.5 ± 2.00 (20.7)	23.8 ± 3.04 (27.2)
Hip joint	Extension/Flexion (°)	44.6 ± 15.8 (35.9)	42.0 ± 9.01 (52.2)
	Abduction/Adduction (°)	14.4 ± 1.79 (16.2)	14.5 ± 4.06 (13.9)
	Lateral/Medial rotation (°)	11.2 ± 2.74 (8.73)	32.2 ± 17.6 (42.5)
Knee joint	Extension/Flexion (°)	63.5 ± 16.7 (57.5)	54.1 ± 17.6 (69.9)
	Abduction/Adduction (°)	23.7 ± 4.47 (28.7)	34.1 ± 19.4 (43.3)
	Lateral/Medial rotation (°)	20.3 ± 5.90 (18.2)	34.0 ± 12.0 (30.6)
Ankle joint	Plantarflexion/Dorsiflexion (°)	125 ± 5.23 (130)	134 ± 22.9 (145)
	Abduction/Adduction (°)	28.7 ± 1.81 (27.9)	19.0 ± 4.46 (16.6)
	Lateral/Medial rotation (°)	24.2 ± 11.5 (22.3)	33.5 ± 6.54 (30.2)
TMP joint	Plantarflexion/Dorsiflexion (°)	78.2 ± 11.1 (68.9)	86.3 ± 12.7 (85.7)
Peak vertical COM velocity (m s^{-1})		0.854 ± 0.0542 (0.832)	0.941 ± 0.0771 (1.01)
Horizontal COM velocity at heel-off (m s^{-1})		0.365 ± 0.0973 (0.257)	0.365 ± 0.222 (0.596)
Dimensionless peak vertical GRF (BW^{-1})		1.345 ± 0.0596 (1.29)	1.569 ± 0.0865 (1.70)

Journal of Experimental Biology • Supplementary information

Dimensionless peak caudal GRF (BW^{-1})	0.363 ± 0.0313 (0.384)	0.317 ± 0.109 (0.253)
Medio-lateral COP range during ascending phase (cm)	29.9 ± 11.5 (20.4)	15.7 ± 8.62 (2.81)

Mean and SD values were calculated using three trials of STS and three trials of STW from one individual. Five additional STW trials were used in analysing kinetic values (i.e., GRFs and COPs), three of which were from a second bird. The values for the exemplar trials used for musculoskeletal simulations are in parentheses. STW durations were obtained from the start of the rising phase to the end of stance phase.

References

Arnold E. M. and Delp S. L. (2011). Fibre operating lengths of human lower limb muscles during walking. *Phil. Trans. R. Soc. B* **366**, 1530–1539. doi: 10.1098/rstb.2010.0345

Dataset 1.

Available for download at
https://journals.biologists.com/jeb/article-lookup/doi/10.1242/jeb.247519#supplementary-data

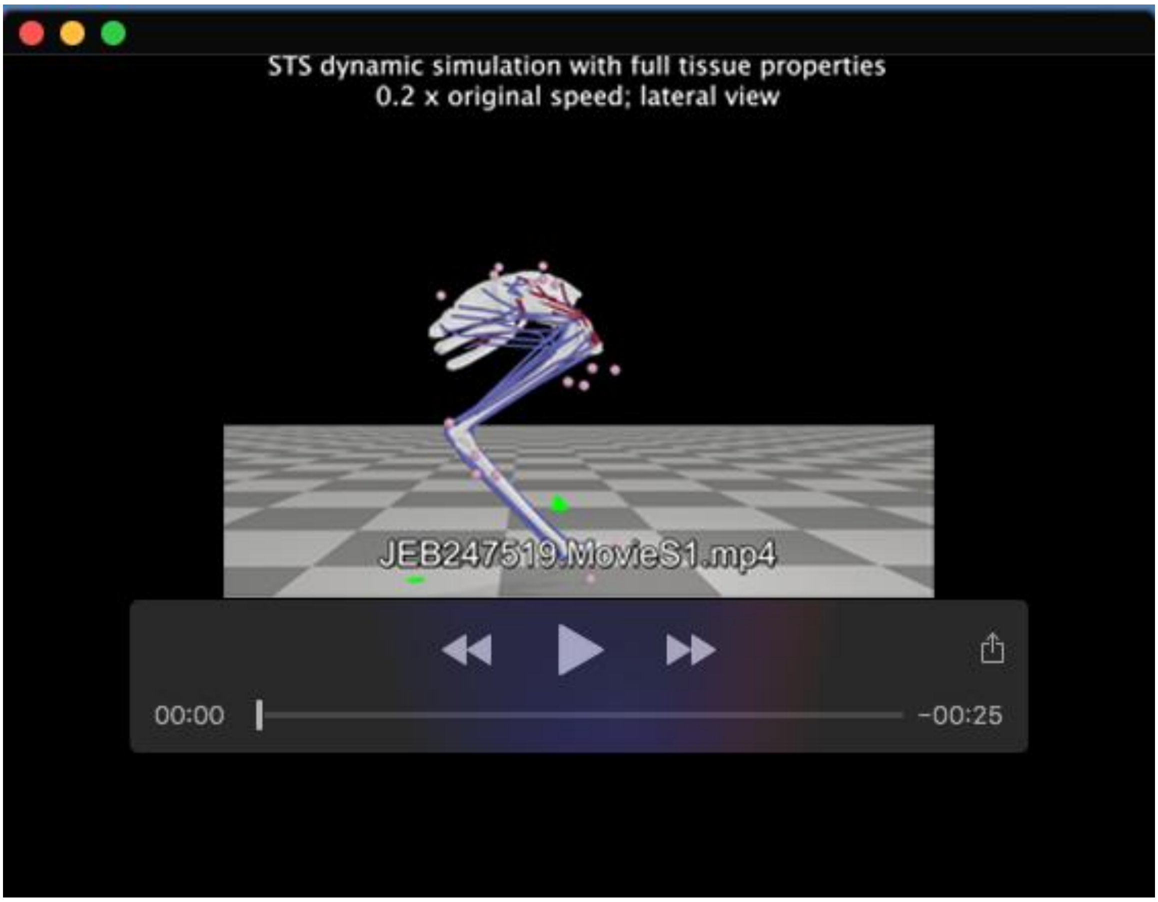

Movie 1. Animation of emu sit-to-stand and sit-to-walk transitions using dynamic simulations with full tissue properties. Movie played at 0.2 x original speed.

Journal of Experimental Biology • Supplementary information

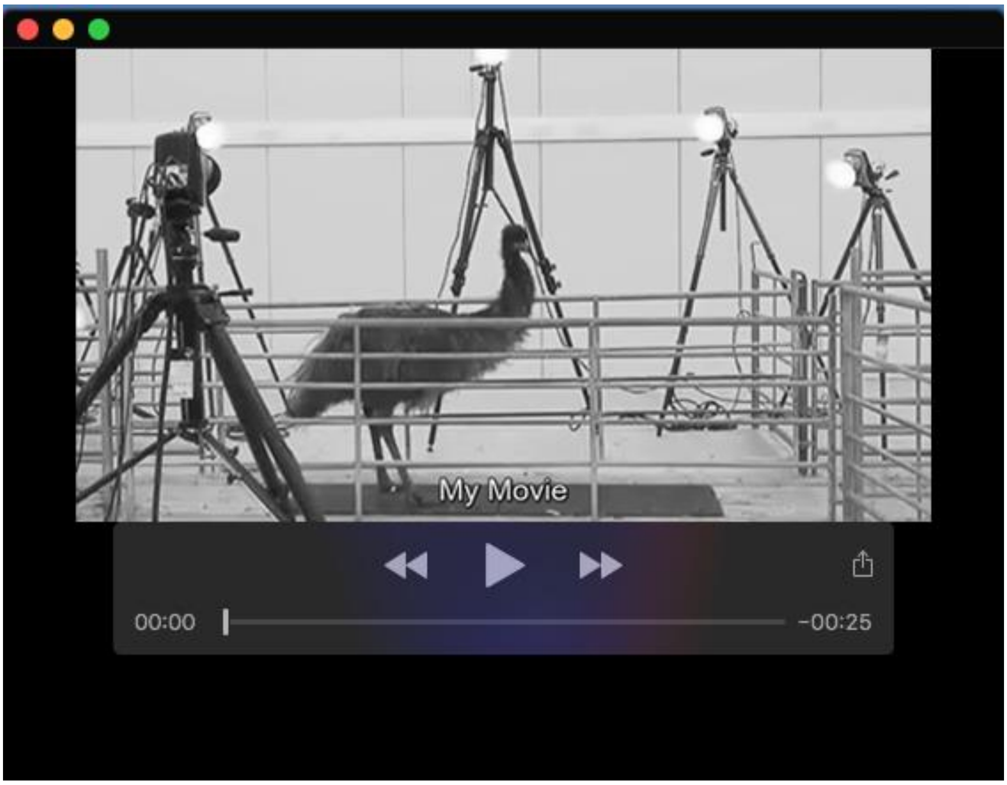

Movie 2. The exemplar trial of an emu sit-to-stand transition.

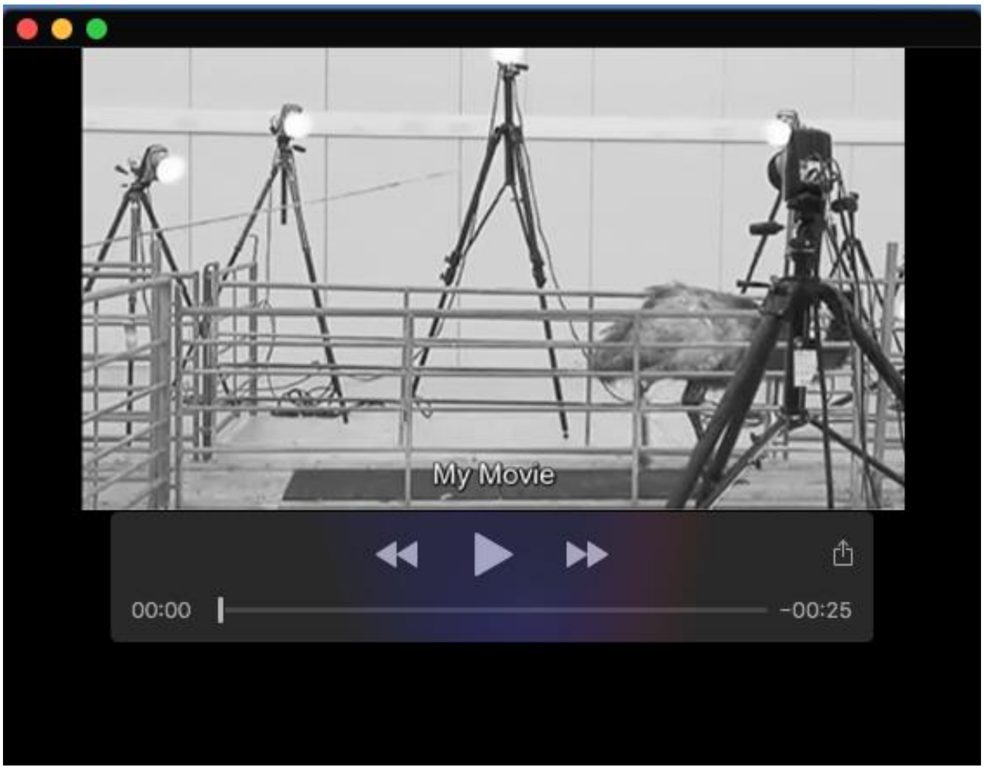

Movie 3. The exemplar trial of an emu sit-to-walk transition.

Journal of Experimental Biology • Supplementary information
